# Auxin Biosynthesis Genes in Allotetraploid Oilseed Rape Are Essential for Plant Development and Response to Drought Stress

**DOI:** 10.3390/ijms232415600

**Published:** 2022-12-09

**Authors:** Mengyu Hao, Wenxiang Wang, Jia Liu, Hui Wang, Rijin Zhou, Desheng Mei, Li Fu, Qiong Hu, Hongtao Cheng

**Affiliations:** Oil Crops Research Institute of Chinese Academy of Agricultural Sciences/Key Laboratory for Biological Sciences and Genetic Improvement of Oil Crops, Ministry of Agriculture and Rural Affairs, Wuhan 430062, China

**Keywords:** auxin biosynthesis, drought resistance, branch angle, YUC, TAR, *B. napus*

## Abstract

Crucial studies have verified that IAA is mainly generated via the two-step pathway in Arabidopsis, in which tryptophan aminotransferase (TAA) and YUCCA (YUC) are the two crucial enzymes. However, the role of the *TAA* (or *TAR*) and *YUC* genes in allotetraploid oilseed rape underlying auxin biosynthesis and development regulation remains elusive. In the present study, all putative *TAR* and *YUC* genes were identified in *B. napus* genome. Most *TAR* and *YUC* genes were tissue that were specifically expressed. Most YUC and TAR proteins contained trans-membrane regions and were confirmed to be endoplasmic reticulum localizations. Enzymatic activity revealed that YUC and TAR protein members were involved in the conversion of IPA to IAA and Trp to IPA, respectively. Transgenic plants overexpressing *BnaYUC6a* in both *Arabidopsis* and *B. napus* displayed high auxin production and reduced plant branch angle, together with increased drought resistance. Moreover, mutation in auxin biosynthesis *BnaTARs* genes by CRISPR/Cas9 caused development defects. All these results suggest the convergent role of *BnaYUC* and *BnaTAR* genes in auxin biosynthesis. Different homoeologs of *BnaYUC* and *BnaTAR* may be divergent according to sequence and expression variation. Auxin biosynthesis genes in allotetraploid oilseed rape play a pivotal role in coordinating plant development processes and stress resistance.

## 1. Introduction

Auxin is a plant hormone that has attracted tremendous amounts of research on its roles in the development, biosynthesis, polar transport, as well as interactions with other phytohormones [1]. Indole-3-acetic acid (IAA) has always been considered a predominant form of auxin in plants. Different aspects of plant development processes, such as seedling growth, embryogenesis and the gravitropism response, are associated with auxin [2]. Plant growth regulation is mainly determined by local auxin concentrations, which are generated through de novo biosynthesis, polar transport and conjugation reactions [3]. As the major plant growth hormone, auxin also plays diverse role in agriculture, such as fruit production, flower development, branching and leaf senescence [4]. Understanding auxin biosynthesis and physiological function has been greatly enriched with the development of genetic, biochemical and molecular studies.

Due to the existence of multiple pathways, auxin biosynthesis has always been regarded as a complex process. Auxin de novo synthesis was considered to include tryptophan-dependent and -independent pathways [5]. Tryptophan-dependent pathways can be divided into four pathways, including indole-3-pyruvic acid (IPA), indole-3-acetaldoxime (IAOx), tryptamine (TAM) and indole-3-acetoamide (IAM) [6]. TAAs (tryptophan aminotransferase) and YUCs (family of flavin monooxygenases) are two crucial enzymes for auxin synthesis and have been placed on the IPA and TAM pathways, respectively, for long time [1]. A breakthrough was made in a two-step pathway of IAA biosynthesis from tryptophan in *Arabidopsis*. It was shown that TAAs and YUCs acted in the IPA pathways together, and YUCs functioned downstream of TAAs [7,8,9,10,11]. In the first reaction, tryptophan is converted to indole-3-pyruvic acid (IPA), and in the second step, IPA is converted to IAA. Using chemical biology approaches, L-amino-oxyphenyl propionic acid (AOPP) and L-Kynurenine were identified to be inhibitors of TAA1 aminotransferase activity [12]. Moreover, yucasin was discovered to be an inhibitor for YUC flavin-containing monooxygenases [13]. Auxin biosynthesis inhibitor L-Kynurenine and yucasin can be used to modulate plant auxin biosynthesis and growth temporally [4].

The abnormal expression of *TAAs* or *YUCs* in several species results in the alternation of auxin levels and in corresponding developmentally abnormal defects. *TAA1* is identified by analyzing mutants for shade-avoidance, ethylene-insensitive and auxin transport inhibitor responses [9,14,15], functioning at the first step of auxin synthesis, which converts Trp to IPA. Four closely related genes of *TAA1* (*TAR1, 2, 3, 4*) were identified in Arabidopsis [14]. The inactivation of *TAA1* results in auxin deficiency and development defects, and these defects can be fixed by supplying exogenous auxin in a medium [4,9]. Mutants of *TAA1* genes in other species also cause decreased IAA levels and related development defects. Unlike *taa1* mutations in Arabidopsis, mutations in a *TAA1* ortholog in maize display dramatic effects on vegetative and reproductive development [16]. The rice gene *FIB* (*fish bone*) encodes an ortholog of *TAA* genes, and the loss of *FIB* function results in pleiotropic abnormal phenotypes and a reduction in internal IAA levels [17]. However, mutations in a homolog of *TAR2* leads to increased auxin levels and elongated seminal roots in *Brachypodium* [18]. The overexpression of *YUCCAs* in Arabidopsis or rice results in high levels of free IAA, therefore generating auxin and overproducing phenotypes [19,20,21]. In other plant species, orthologs of *YUCCA* also appear to be involved in auxin synthesis based on their effects on plant development [21,22,23].

Although considerable progress on auxin synthesis have been made in several species of plants, little information has been obtained about the molecular mechanism of auxin synthesis in *B. napus*. Auxin concentrations during the progression of embryogenesis are progressively increased in *B. napus* and are correlated with the expression levels of genes in auxin biosynthesis pathways [24]. The *TTG2* gene in *B. napus* regulates trichome development and increases plant sensitivity to salt stress by suppressing the expression of auxin biosynthesis genes [25]. Through QTL-seq analysis, *BnaYUC6* was identified as a candidate gene controlling the branch angle [26]. In order to uncover the detailed role of auxin biosynthesis in plant development and environmental stress responses in oilseed rape, we identified all *YUC* and *TAR* genes in the *B. napus* genome and characterized their expression pattern, cellular localization and functional roles. The major findings in this study provide comprehensive information of the conserved auxin biosynthesis pathway in *B. napus* and highlight the pivotal role of these genes in plant development and environmental stress resistance.

## 2. Results

### 2.1. Identification and Phylogenetic Analysis of BnaTAAs and BnaYUCs in B. napus

**Figure 1:**
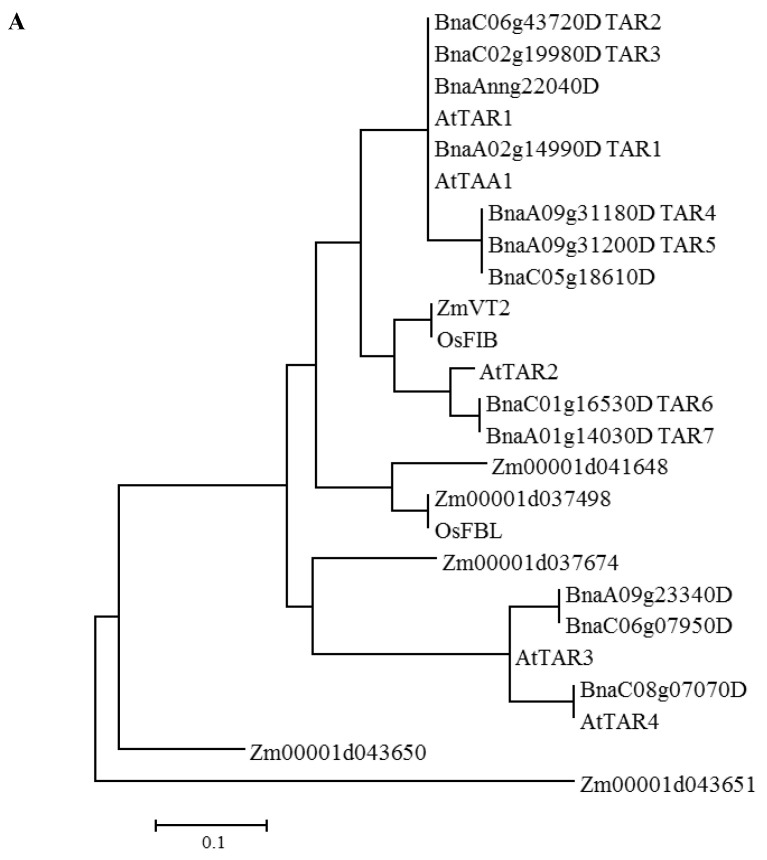

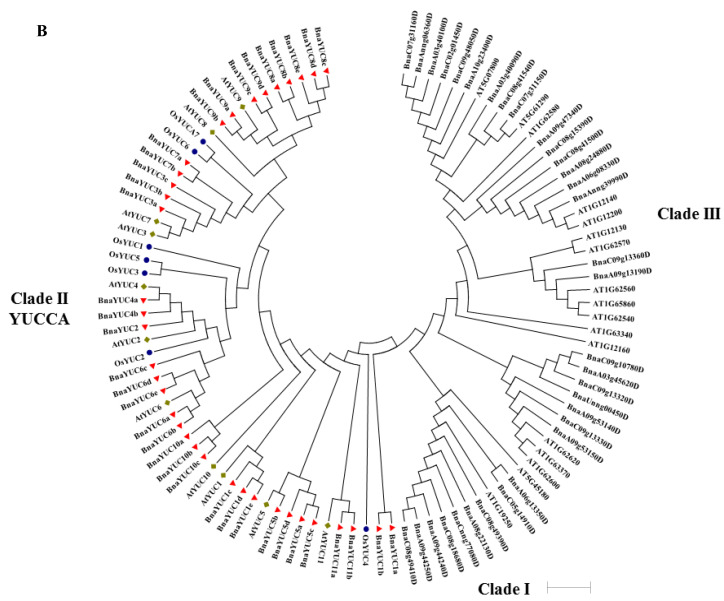
Phylogenetic analysis of TAR and YUC proteins: (**A**) TAR proteins from Arabidopsis, rice and rapeseed were used to construct the phylogenetic tree by using the neighbor-joining algorithm with 1000 replications. (**B**) FMO-like protein from rapeseed and Arabidopsis was used to construct the unrooted phylogenetic tree with rice YUC proteins. Proteins were divided into three clades, and clade II FMOs were in the *YUC* gene family. YUC proteins from rice, Arabidopsis and rapeseed were marked with circles, squares and triangles. The phylogenetic tree was constructed using the neighbor-joining algorithm with 1000 replications. Bars indicate 0.1 aa substitution per residue.

Twelve putative homologs of TAA/TAR proteins were identified in the *B. napus* genome database. Phylogenetic analysis divided these TAA/TAR proteins into three separate clades (Figure 1A). AtTAA1, AtTAR1 and six BnaTAR homologs were classified into the first clade; AtTAR2, BnaTAR7 and BnaTAR8 were grouped into the second clade; and two TAA/TAR proteins from monocot plants Zmvt2 and OsFIB were classified into the third group (Figure 1A).

An HHMER search using FMO-domain PF03110 as a query against the *B. napus* protein database identified seventy-eight FMO (flavin-containing monooxygenase) proteins. After checking with an Interpro scan, eight proteins without complete FMO domains were excluded. FMOs from *B. napus*, rice and Arabidopsis could be clearly divided into three independent groups, including one group defined as YUCs (Figure 1B). Thirty-eight *YUC* genes in *B. napus* were designated as *BnaYUC* and were named according to the order of homologous genes in Arabidopsis. Further analysis divided the YUCs in *B. napus* into different groups, with each one including five to eight members (Figure S1). Tandem duplicated *YUCs* in the oilseed rape genome were distinctly assigned to one group. To obtain detailed gene structure information, we compared the coding sequence with the genomic sequences of all *YUCs* in oilseed rape, and 1 to 4 introns were observed among the *YUC* genes (Figure S1). *BnaYUC* genes clusters with similar structures were grouped together. The YUC proteins belong to the FMO family with conserved motifs, including FMO-identifying and FAD- and NADPH-binding motifs (Figure S2A). Using MEME tools, all these conserved motifs could be found in YUC proteins in oilseed rape (Figure S2B).

### 2.2. BnaYUCs and BnaTAAs Exhibit Tissue-Specific Expression Patterns

**Figure 2:**
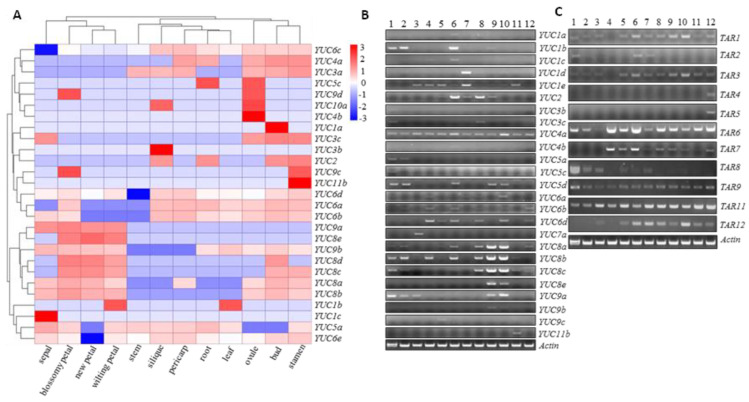
Expression patterns of *BnaYUC* genes in twelve different tissue samples: (**A**) Tissues used for expression profiling are indicated at the bottom of each column. Color scale bars at the top of map represent log2 transformed FRKM values. Values of 3, 0 and −3 represent positive, zero and negative expression, respectively. The genes are on the right of the expression bar. (**B**,**C**) Tissue expression pattern validated by RT-PCR. Tissues used for expression profiling are indicated at the top of each column. 1, young root; 2, hypocotyl; 3, cotyledons; 4, stem; 5, leaf; 6, small bud; 7, big bud; 8, pistil; 9, stamen; 10, petal; 11, flower stalk; 12, silique. Genes are on the left of expression bar. Actin gene was amplified with 27 cycles. Other genes were amplified with 35 cycles.

The FPKM values of *BnaYUCs* in Zhongshuang 11 were extracted from the RNA-seq results (Figure 2A). The transcript values of nine *BnaYUC* genes were zero in twelve tissue samples, and nine of thirty-six *BnaYUCs* genes were only expressed in one or two specific tissues (Figure 2A). To ascertain the detailed expression level of *YUCs* and *TAAs* in various tissues in *B. napus*, we performed a semi-RT-PCR (semi-quantative reverse transcription PCR) analysis with gene-specific primers. The expression levels of *BnaYUCs* and *BnaTARs* in four seedling tissue samples and eight tissue samples at the adult stage were compared, and the results show that most *BnaYUC* genes were specifically expressed tissues (Figure 2B). *BnaYUC4a* was detected in all tissue samples, but expression levels in different tissues were also variant (Figure 2B). Although *BnaYUC1e*, *6b* and *6d* transcripts were detectable in most samples, they reached a clearly higher level in tissues in particular (Figure 2B). The remaining *YUC* genes showed an overall tissue-specific expression pattern. With respect to sampling stage, *BnaYUC3b*, *5a*, *5c* and *7a* were only partially expressed in seedling samples, including cotyledons, young leaves and roots (Figure 2B). Faint expression of *BnaYUC1a* and *4b* was observed only in young flower buds. *BnaYUC9c* and *11b* were detected to be expressed only in leaves and flower stalks, respectively (Figure 2B). Predominant expression of *BnaYUC8a*, *8b*, *8c*, *8e* and *9a* was detected in most floral organs (Figure 2B). 

Only twelve *BnaTARs* in *B. napus* were recognized to be homologs of *AtTAA1* or *AtTAR1*. With the exception of *BnaTAR4* and *5*, which were specifically expressed in young silique, other *BnaTAR* genes were detected in more than two tissue samples (Figure 2C). For example, a transcript of *BanTAR2* was detected in young seedling and flower samples. It should be noted that the expression of *BnaTAR9* and *BnaTAR11* was perceived in all investigated tissue samples (Figure 2C). Therefore, both *BnaYUC* and *BnaTAR* genes possessed diverse but highly specific expression patterns, which may determine spatial and temporal auxin biosynthesis, which is in charge of certain physiological processes.

### 2.3. Expression of BnaTARs and BnaYUC Genes under High Temperature

**Figure 3:**
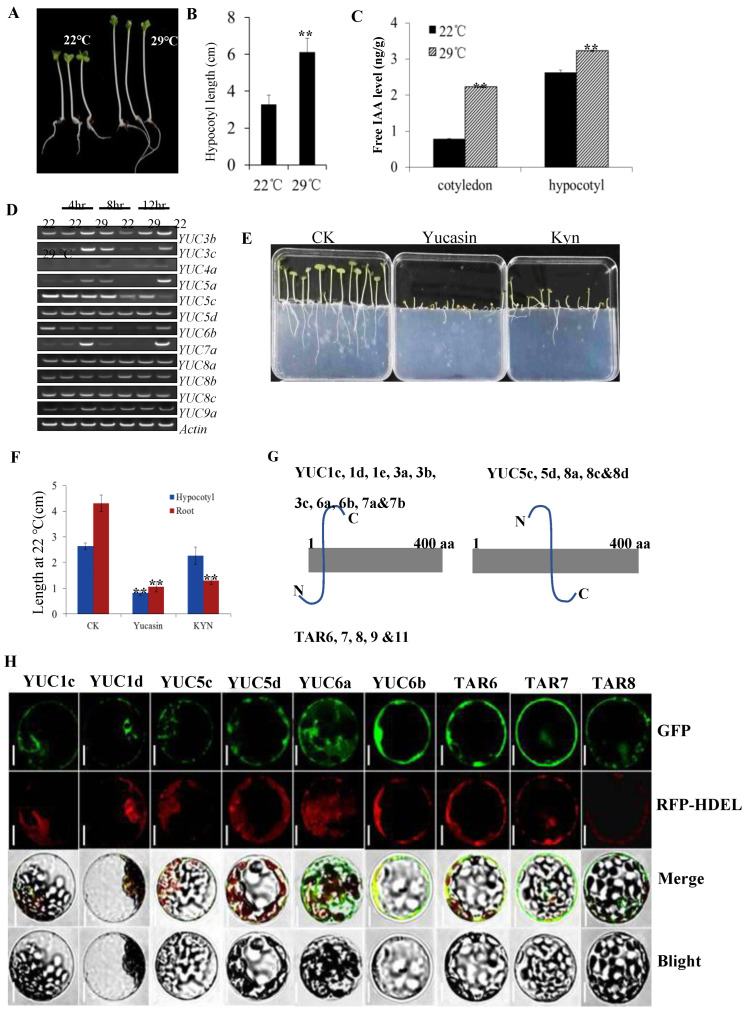
Seedlings treated with auxin biosynthesis inhibitor or different temperatures, and subcellular analysis of auxin biosynthetic genes in oilseed rape. (**A**) Seedling grown for 5 days at 22 °C and 29 °C. (**B**) The seedlings displayed elongated hypocotyls at high temperatures. Asterisks indicate significant differences from the control (** *p* < 0.01, Student’s *t* test). (**C**) Free IAA levels in the cotyledons and hypocotyl at 5 days. Asterisks indicate significant differences from the control (** *p* < 0.01, Student’s *t* test). (**D**) Higher temperatures induced *YUC* gene expression in *B. napus.* (**E**) Effects of yucasin and kynurenine on *B. napus* seedlings grown in MS medium with 100 μM concentrations for 5 days. (**F**) Effects of yucasin and kyn on hypocotyl elongation of seedlings. Asterisks indicate significant differences from the control (** *p* < 0.01, Student’s *t* test). (**G**) Trans-membrane structure analysis of TAR and YUC protein. (**H**) Localization of auxin biosynthetic proteins by transient expression in Arabidopsis protoplast. Co-expression of ER localized marker HDEL-RFP with BnaTAR-GFP or BnaYUC-GFP proteins were analyzed. Bars = 10 μm.

The hypocotyl of oilseed rape seedlings was elongated more under high temperatures compared with normal conditions (Figure 3A,B). Studies on Arabidopsis substantiate that the elongation of hypocotyl is closely linked with increased auxin biosynthesis. To understand whether the IAA biosynthesis pathway is changed by temperature, free IAA levels of hypocotyl and cotyledon under different treatments were measured. We observed that the free IAA level was increased in the hypocotyl and cotyledons under higher temperatures compared with normal conditions (Figure 3C). To check whether *BnaYUC* genes contribute to the alternation of IAA levels, the expression of *BnaYUC* genes in tissues of plants under different temperatures was analyzed. After being moved to 29 °C for 4 h, transcripts of *BnaYUC3c*, *5a* and *7a* were enriched significantly compared with samples at 22 °C (Figure 3D). The expression of Bna*YUC3c*, *5a*, *6b* and *7a* was also significantly increased after being treated with 29℃ for 12 h (Figure 3D). These results indicate the possible link of more highly expressed auxin biosynthesis genes with increased IAA levels in elongated hypocotyl under higher temperatures in oilseed rape. 

### 2.4. Kynurenine and Yucasin Suppressed Auxin Biosynthesis in Oilseed Rape 

According to previous studies, kynurenine and yucasin are two chemicals inhibiting the activity of AtTAAs and AtYUCs in *Arabidopsis* [12,13]. To check whether these inhibitors suppress auxin biosynthesis in *B. napus*, seedlings were treated with kynurenine or yucasin at different time points. After treating with kynurenine (50 μM) and yucasin (100 μM) for six days, the lengths of both the hypocotyl and root were significantly inhibited (Figure 3E,F). The expression level analyzed by RT-PCR shows that Kynurenine and yucasin treatment decreased the expression of *BnaYUC1b*, *5d*, *8b*, *BnaTAR3*, *7* and *11* in the root (Figure S3A). The transcripts of *BnaYUC8a*, *8b* and *BnaTAR2* were upregulated after treatment with yucasin or Kynurenine for 8 hr in the aerial part (Figure S3B). Expression levels of *BnaYUC4a* were significantly enhanced under the yucasin treatment (Figure S3B). 

### 2.5. Subcellular Localization of BnaTAR and BnaYUC Proteins

The trans-membrane prediction of TAR/YUC proteins in oilseed rape by the TMHMM tool showed that most BnaYUC proteins possess one trans-membrane domain (TMD) (Figure S4). One typical trans-membrane domain was found in the BnaTAR6, 7, 8 and 9 proteins (Figure S4). The TMD regions of BnaTAR6, 7, 8, 9 and 11 and of BnaYUC1c, 1d, 1e, 3a, 3b, 3c, 6a, 6b, 7a and 7b were localized in the N-terminal (Figure 3G). The TMD region of the remaining five proteins, YUC5c, 5d, 8a, 8c and 8d, was localized in the middle of protein (Figure 3G). To ascertain whether TMD location affects subcellular localization, some BnaYUC and BnaTAR proteins with or without TMD regions were selected to perform subcellular localization analysis. To determine detailed subcellular localization, each BnaTAR and BnaYUC protein was fused with the C-terminal of the GFP fluorescent tag. All these fused proteins were co-expressed with the ER marker RFP-HDEL and were visualized by confocal microscopy. BnaYUC1c, 1d, 5c, 5d, 6a, 6b and BnaTAR6, 7 and 8 were co-localized with the ER-marker GFP–HDEL (Figure 3H).

### 2.6. Enzymatic Activity of BnaYUC and BnaTAA Proteins

**Figure 4:**
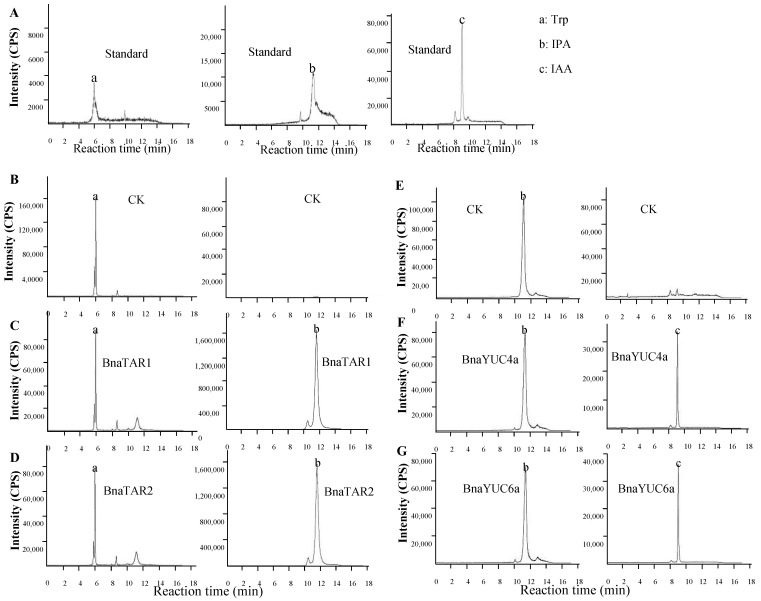
Enzymatic analysis of BnaYUCs and BnaTAR proteins: (**A**) Chromatogram of Trp, IPA and IAA authentic standard. (**B**) Enzyme reaction mixture for Trp with protein tag. (**C**,**D**) Enzyme reaction mixture for Trp with purified BnaTAR1 and BnaTAR2 protein, respectively. (**E**) Enzyme reaction mixture for IPA with protein tag. (**F**,**G**) Enzyme reaction mixture for IPA with purified BnaYUC4a and BnaYUC6a protein, respectively. Arrows indicate the Trp (a), IPA (b) and IAA (c) peaks.

BnaTAR1 and BnaTAR2 were expressed in *Escherichia coli* BL21 (DE3) cells (Figure S5). Purified recombinant proteins of BnaTAR1 and BnaTAR2 obtained with His-tag were used to perform an enzyme assay in a PBS buffer containing Trp, PLP (Pyridoxal-5-phosphate monohydrate) and Sodium Pyruvate. We then confirmed the product and zymolyte of this reaction with LC/MS, and a product peak of IPA could be detected in both reaction systems with BnaTAR1 and BnaTAR2. A peak was not observed in the reaction system that only contained an empty His-tag (Figure 4A–D). This result suggests that purified His-BnaTAR1 and His-BnaTAR2 can convert the L-Trp to IPA (Figure 4A–D). After that, we examined the enzyme activity of BnaYUCs in vitro, with the reaction buffer changed to IPA, FAD and NADPH, and the reaction products were detected with LC/MS. The peak of IAA was observed in the product of His-BnaYUC6a as well in that of His-BnaYUC4a (Figure 4F,G). However, no IAA was found in the reaction with the purified His-tag, which was used as the negative control (Figure 4E). These results indicate that the BnaYUC6a and BnaYUC4a proteins possess the ability to convert IPA to IAA.

### 2.7. Plants Overexpressing BnaYUC Genes Showed an Auxin-Overproduced Phenotype and Decreased Branch Angle

**Figure 5:**
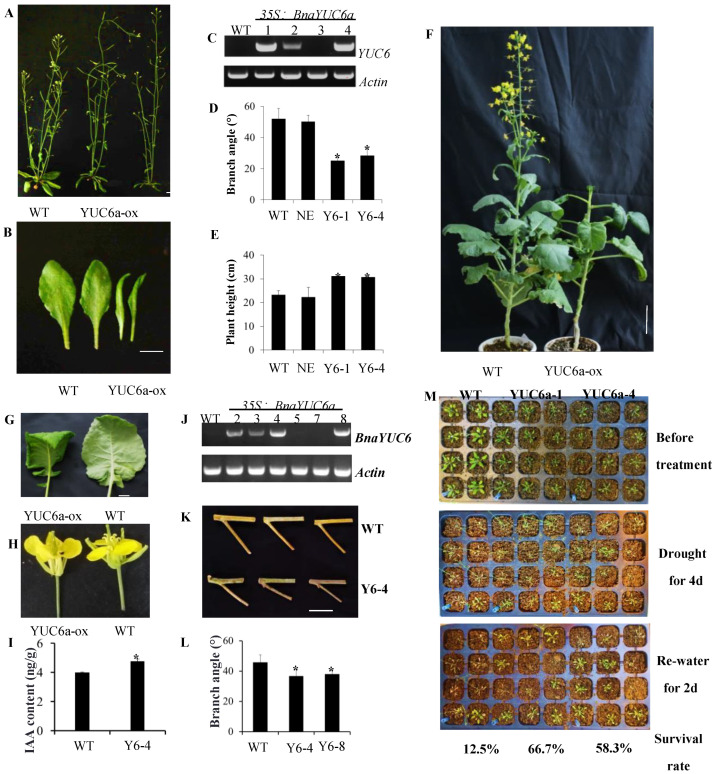
*BnaYUC6a*-overexpressing plants changed in plant architecture and drought resistance. (**A**) Phenotype of BnaYUC6a-ox transgenic and wild type in Arabidopsis. Bar = 1 cm. (**B**) BnaYUC6-ox transgenic plants display narrow and curled leaves in Arabidopsis. Bar = 1 cm. (**C**) Expression level detection of BnaYUC6a-ox transgenic plants. (**D**,**E**) Overexpressed *BnaYUC6a* significantly decreased plant branch angle and increased plant height.Asterisks indicate significant differences from the control (* *p* < 0.05, Student’s *t* test). (**F**) Overexpression of *BnaYUC6* in oilseed rape showed late flowering time with curled leaves (**G**) and large flowers (**H**). Bar = 5 cm (**F**) and Bar = 1 cm (**G**,**H**). (**I**) Auxin concentration in BnaYUC6-ox and wild type in oilseed rape. Asterisks indicate significant differences from the control (* *p* < 0.05, Student’s *t* test). (**J**) Expression analysis of *BnaYUC6* in oilseed rape. BnaYUC6a-ox transgenic plants decreased branch angle (**K**,**L**)Asterisks indicate significant differences from the control (* *p* < 0.05, Student’s *t* test). Bar = 5 cm. (**M**) Drought stress treatment of BnaYUC6a-ox and wild type. After photographing, plants underwent a drought for 3 days and then were re-watered for 2 days. The survival rate was quantified and is shown at the bottom of the picture.

To investigate the putative role of *BnaYUC* genes in auxin biosynthesis, transgenic plants overexpressing *BnaYUC6a* and *BnaYUC4a* in Arabidopsis under the control of a CaMV35S promoter were generated. Expression level detection showed that *BnaYUC6a* and *BnaYUC4a* were overexpressed in the transgenic Arabidopsis lines (Figure 5C and Figure S6C), and these transgenic lines showed an obvious auxin-overproduced phenotype, such as extremely tall inflorescences with extreme apical dominance and twisted cauline leaves (Figure 5A,B and Figure S6A–C). The overexpression of these two genes significantly decreased the branch angle but increased plant height in Arabidopsis (Figure 5D,E and Figure S6D,E). To further discern the function of *BnaYUC* genes, we obtained *BnaYUC6a*-overexpressing plants in oilseed rape (Figure 5J). BnaYUC6a-ox in oilseed rape (*BnaYUC6a* overexpressing) seedlings also exhibited epinastic cotyledons and narrow leaves with downward curled edges (Figure 5F,G). The flower was comparatively larger than the wild type (Figure 5H). Increased auxin levels were detected in *BnaYUC6a*-overexpressing plants compared with wild-type plants (Figure 5I). The leaf angles of BnaYUC6a-ox plants were significantly larger than that of the wild type (Figure 5G). However, the branch angle of BnaYUC6a-ox plants in oilseed rape were decreased significantly at the adult stage (Figure 5K,L). We also obtained *BnaYUC6*-mutated plants by CRISPR/Cas9. *BnaYUC6a* with another similar copy, *BnaYUC6b*, was mutated after sequence verification (Figure S7A). However, transgenic plants did not exhibit an auxin-deficient phenotype, such as the loss of apical dominance or semi-dwarfism (Figure S7B). As *BnaYUC* genes form one large family in oilseed rape, this result may be caused by functional redundancy among the paralogs.

### 2.8. Ectopic Expression of BnaYUC Genes Leads to Drought Resistance

To assess whether *BnaYUC* genes were involved in drought resistance, we examined the expression level of *BnaYUC* and *BnaTAR* genes with ABA and PEG treatments. The expression of *BnaYUC1b*, *4a*, *5d* and *6a* was induced after ABA and PEG treatment in root samples (Figure S8A). However, we found a transcript of some *BnTAR*s, including *BnaTAR2*, *7* and *11*, which were decreased after both ABA and PEG treatments in root samples (Figure S8A). Increased expression levels of *BnaYUC8a* and *8b* in shoot samples were also observed after ABA and PEG treatment (Figure S8B). In contrast, expression levels of *BnaTAR2* and *7* were suppressed after treatment with ABA or PEG in shoot samples (Figure S8B). Most *BnaYUCs* expression was induced, whereas *BnaTARs* were suppressed after ABA or PEG treatment, suggesting that *BnaYUCs* were more likely to be involved in drought resistance.

**Figure 6:**
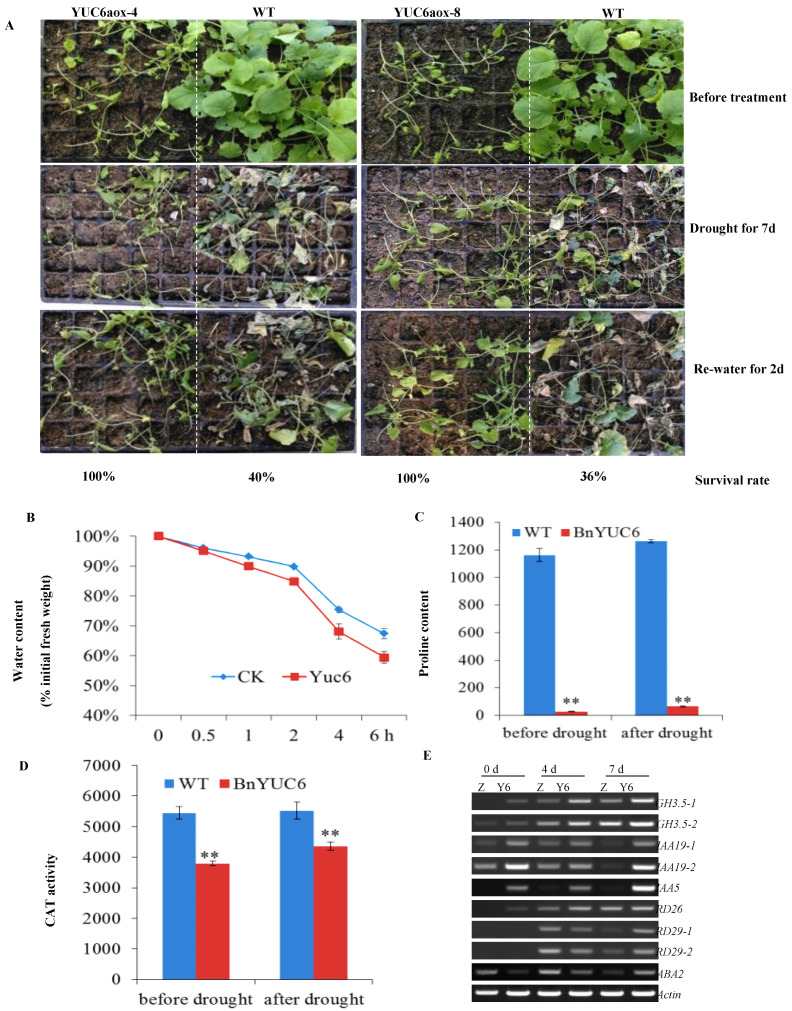
Overexpressed *BnaYUC6a* in *B. napus* increased drought resistance: (**A**) Two BnaYUC6a-ox lines and wild type seedlings were well grown for three weeks under normal conditions and were then photographed (first panel). After drought for seven days, the plants were photographed (two panel). The plants were then re-watered for two days and were photographed (third panel), and the survival rate (percent) in each sample was quantified, as shown at the bottom of the picture. (**B**) Water loss assays. Seedling plants were grown in soil for 4 weeks, and the third to fifth rosette leaves were used. (**C**) Proline content detection. Leaves from four-week-old plants before drought or after drought for 4 days were used to detect proline content. (**D**) CAT activity detection. The sample was selected as a proline test. (**E**) Auxin- and ABA-responsive gene expression in YUC6a-ox and wild-type plants. The sample was harvested before drought or after drought for 4 and 7 days. *Actin* gene was used as the control. All statistical tests were performed with Student’s test. Bars indicate standard error of the mean (** *p* < 0.01).

To confirm the potential function of *BnaYUC* genes in water deficit stress, three-week-old plants of both the *BnaYUC6a*-overexpressing transgenic line and the *BnaYUC4a*-overexpressing transgenic line, as well as the wild-type Arabidopsis, were treated with water deficit stress by withdrawing water for 3 or 4 days after the soil dried completely. Wilting symptoms in wild-type plants were significantly more pronounced than those of the *BnaYUC6a*-overexpressing lines (Figure 5M) and BnaYUC4a-overexpressing line in Arabidopsis (Figure S6F). After re-watering for two days, the survival rates of *BnaYUC6a*-overexpressing lines were 66.7% and 58.3%, the survival rate of the *BnaYUC4a*-overexpressing lines was 64%, and both of them were significantly higher than the percentages of 12.5% and 20% for the wild type (Figure 5M, Figure S6F). Furthermore, *B. napus* transgenic plants overexpressing *BnaYUC6a* were also subjected to water deficit conditions for the assessment of drought resistance. After six days without water supplement, more severe wilting symptoms were observed in the wild type than in the *BnaYUC6a*-overexpressing lines (Figure 6A). The difference in wilting severity was more pronounced after water recovery (Figure 6A). After re-watering for two days, 100% of the *BnaYUC6a*-overexpressing lines survived, whereas less than 40% of the wild type plants survived (Figure 6A).

### 2.9. Overexpression of BnaYUC6a-Activated Genes Involved in Drought Resistance 

To evaluate the resistance level of *BnaYUC6a*-overexpressing lines to water deficiency, the water loss ratio was assessed in Arabidopsis. Surprisingly, BnaYUC6a-ox plants at the rosette stage lost water more quickly compared with the wild type (Figure 6B). The catalase (CAT) activity was much lower in transgenic plants than in the WT both before and after drought treatment (Figure 6D). Moreover, it was found that *BnaYUC6a*-overexpressing plants maintained a lower content of free proline than the WT, even after 4 days of drought stress (Figure 6C). This contradictory result may be due to the abnormal development of leaves in *BnaYUC6a*-overexpressing plants compared with the wild type. No expression level or very low expression levels of drought-responsive genes, including *RD26*, *RD29* and *ABA2*, were observed both in the *BnaYUC6a*-overexpressing lines and in the wild type before drought treatment (Figure 6E). However, the transcript level of these genes was significantly increased in *BnaYUC6a*-overexpressing lines compared with the wild type after water deficiency treatment for 7 days (Figure 6E). Some of the auxin-responsive genes, including *IAA19-2*, *IAA5* and *GH3.5-1*, were also significantly increased in *BnaYUC6*-overexpressing lines compared with the WT (Figure 6E).

### 2.10. Mutation of TAR Homologs in B. napus Caused Developmental Defect

**Figure 7:**
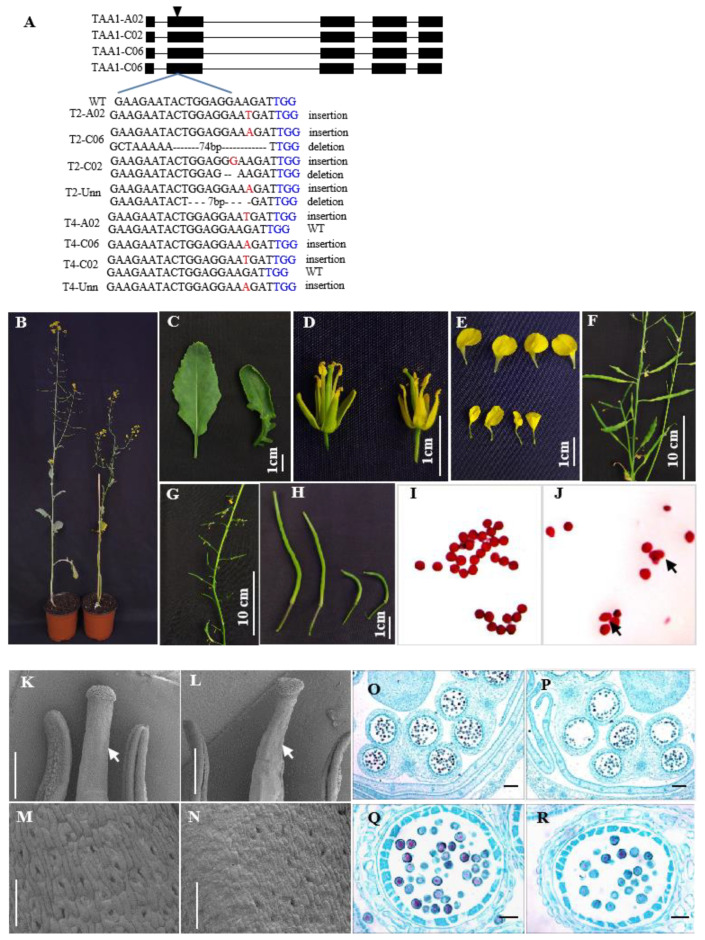
Phenotype of *Bntar* mutant created by CRIPSR/Cas9: (**A**) Sequencing results of two *Bntar* mutants. Letters in black and blue indicate target sequences and PAM; red color and hyphen in the target sequences indicate insertions and deletions, respectively. (**B**) Phenotype of *Bntar* mutant (right side) and wild type (left side). (**C**) Leaf shape of *Bntar* mutant (right side) and wild type (left side). (**D**) Flower bud of Bntar mutant (right side) and wild type (left side). (**E**) Petal shape of *Bntar* mutant (upper side) and wild type (lower side). (**F**) Wild type and (**G**) *Bntar* mutant, showing images of silique shapes in main inflorescence. (**H**) Enlarged picture of pod at 10 days after flowering; wild type (left side) and *Bntar* mutant (right side). (**I**,**J**) Pollen stained by Alexander stain solution: wild type (**I**) and *Bntar* (**J**), arrows indicate enlarged pollen. Scanning electron microscopy image of wild-type (**K**) and *Bntar* (**L**) pistils and stamens. Arrows indicate shrinking sigma. Enlarged images of stigma of wild type (**M**) and *Bntar* (**N**). (**O**–**R**) Cross-sections of flower buds of wild type (**O**,**Q**) and *Bntar* (**P**,**R**). (**Q**,**R**) Images enlarged by one microsporangium. Bar = 2 μm (**E**,**F**) and Bar = 10 μm (**G**,**H**).

To address the full auxin biosynthesis pathway, four homologs of *AtTAA1* in oilseed rape were simultaneously mutated by CRISPR/Cas9. Transgenic plants with four homologs simultaneously knocked out were identified (Figure 7A). Homozygous mutants with four *BnaTAR* homologs mutated showed dwarfism with rolled branches and increased branch angles (Figure 7B). Smaller and rolled leaves were observed in the mutant plants (Figure 7C). At the reproductive phase, *BnaTAR* homozygous mutants formed smaller flowers with bent gynoecia (Figure 7D). Flower petals exhibited smaller and cupped shapes compared with the wild type (Figure 7E). Most *BnaTAR* homozygous mutant siliques were abnormally developed and hardly underwent seed setting (Figure 7F–H). Irregular pollen shapes were observed in *BnaTAR* homozygous mutants after staining with Alexander staining solution (Figure 7I,J). The scanning electron microscopy results show that the pistils of *BnaTAR* homozygous mutants were wrinkled with small and abnormally arranged guard cells (Figure 7K–N). Moreover, less mature pollen was observed in the anthers of *BnaTAR* homozygous mutants (Figure 7O–R). After being transplanted in the field, the mutants exhibited a severe dwarfism phenotype and died before flowering (Figure S9). We then isolated *BnaTAR* mutants with three mutated homologs, and the plants also showed dwarfism in the field (Figure S10A). However, the mutants could grow into normal plants with extremely lower growth speeds. Mutants displayed abnormal stem development, in which branches or stems adhered together (Figure S10B,C). Rosettes and cauline leaves in Bnatar mutants also developed abnormally with bulging leaf veins (Figure S10D,E).

## 3. Discussion

### 3.1. Large Numbers of BnaYUC and BnaTAR Genes Exist in the B. napus Genome

Flavin-containing monooxygenases (FMOs) in plants form one large, expanded family. Genetic and biochemical studies have revealed that some plant FMOs can catalyze specific steps in the biosynthesis of auxin [19,27,28]. Eleven of twenty-nine putative FMOs belong to *YUC* genes, and most of them have been demonstrated to be involved in the biosynthesis of the essential hormone auxin in *Arabidopsis*. In our study, seventy FMO members were identified in *B. napus*, and thirty-six of them belong to the *YUC* gene family. The FMO-identifying motif, FAD- and NADP-binding motifs, the WL (I/V) VATGENAE motif and the F/LATGY motif were identified in all BnaYUC proteins, suggesting similar biochemical YUC protein function between *Arabidopsis* and *B. napus*. Twelve TAR proteins were found in the B. napus genome, and phylogenetic analysis revealed that these proteins have close relationships with AtTAA1/TAR1/TAR2 proteins. According to previous results, the function of *AtTAA1*/*TAR*1/*TAR2* is overlapped in auxin biosynthesis [9]. Many more *BnaYUCs* and *BnaTARs* exist in *B. napus*, suggesting that the function of these genes is redundant in auxin biosynthesis as well as in other biological processes. Although *AtYUCs* and *AtTARs* have been shown to be involved in the two-step pathway of IAA biosynthesis in Arabidopsis, little information of these two gene families has been obtained in other species. 

### 3.2. The Expression Patterns of Most YUC Genes in B. napus Are Highly Tissue-Specific

There are eleven *YUC* and three *TAA1*/*TAR* genes in the Arabidopsis genome. Expression analysis revealed that *TAA1* was expressed in most plant tissues, such as in cotyledons, young leaves and vascular tissue in shoots and roots [9]. Similar to the *TAA1* family in *Arabidopsis*, rice *FIB*, which is homolog of *TAA1*, also showed extensive expression patterns, including in vegetative shoots and inflorescence apexes, leaf sheaths, young flowers and roots [17]. Different from the expression pattern of *AtTAA* genes, *AtYUC* genes are more spatially and temporally regulated. Relatively high levels of *AtYUC1*, *AtYUC2*, *AtYUC4* and *AtYUC6* genes were detected in young primordia, vascular tissues and flower organs [27]. In Arabidopsis, *YUC* genes appear to be separated into two sets according to the auxin biosynthesis in roots and shoots [29]. *AtYUC 3*, *5*, *7*, *8* and *9* are considered to be responsible for auxin production in roots, whereas *AtYUC1*, *2*, *4* and 6 mainly function in shoots [30]. More specific expression patterns of *BnaYUC* genes were observed among twelve tissues, including in vegetative and reproductive stages, compared with *BnaTAR* genes in *B. napus*. Most *BnaTAR* genes were expressed in all tissues, whereas *BnaTAR4* and *BnaTAR5* were only detected in siliques. Six *BnaYUC* genes were found to be expressed in only one specific tissue. Other *YUC* genes showed expression in two or more tissues. Within the auxin IPA biosynthesis pathway, *YUC* genes catalyze the rate-limiting step. The strict expression pattern of *YUC* genes may lead to spatiotemporal IAA biosynthesis, thereby affecting specific aspects of plant growth and development.

### 3.3. BnaYUC and BnaTAR Proteins Possessed Auxin-Biosynthesis-Related Enzyme Activity

FAD and NADPH were used as cofactors together with molecular oxygen substrate in the FMO reaction. YUC proteins belong to large FMO family containing two GXGXXG motifs, which may function as the binding site for FAD and NADPH [31]. To determine whether the IPA was the substrate of YUC proteins, the enzymatic assays were conducted in vitro in Arabidopsis [7,32]. The recombinant YUC2 protein expressed in and purified from *E. coli* with the GST could convert IPA to IAA in vitro [7]. Moreover, Arabidopsis YUC4, with two alternative splice variants localized in the endoplasmic reticulum and cytosol, was able to convert IPA to IAA [32]. In our study, three YUC proteins (BnaYUC1e, 4a and 6) were expressed in *E. coli* and showed the use of IPA as a substrate. These results suggest that YUC proteins from *B. napus* are also able to convert IPA to IAA. TAAs and YUCs were unambiguously placed in the same Trp-dependent auxin biosynthesis pathway in previous results [6]. TAAs acted in the first reaction, which converted tryptophan to indole-3-pyruvic acid (IPA). Recombinant TAA1 proteins with the tag catalyzed the PLP-dependent transaminase reaction in vitro [9,14]. Besides TAA1 in *Arabidopsis*, only one more TAR from peas was shown to qualitatively convert Trp to IPA [33]. In this study, the recombinant TAA protein from *B. napus* was shown to be able convert Trp to IPA in vitro. The specific enzymatic activity of YUCs and TARs from *B. napus* may reveal the conservation of the auxin biosynthesis pathway between the allopolyploid and diploid species during the evolution procedure.

### 3.4. Subcellular Localization of Auxin Biosynthesis Genes in B. napus

The AtYUC6 protein was shown to have a non-plastidial subcellular localization in an unidentified intracellular compartment [20]. Auxin biosynthetic protein AtYUC4 has two splice variants, which were shown to be localized in the endoplasmic reticulum and cytosol [32]. Several YUC proteins in maize were found to be localized in the endoplasmic reticulum [34]. Arabidopsis TAA1 functions at the first step of auxin synthesis, converting Trp to IPA, and was predicted to be cytosolic localized. In our study, BnaTAR1, 6, 9 and 10 were localized in the cytosol. Proteins BnaYUC1e, 4a and 6 possessing the TMD domain were localized in cytosol, whereas other BnaYUC proteins, BnaYUC8a, 10a and 11b, were ER localized. The ER localization of auxin biosynthetic proteins may be associated with the other part of auxin action [34]. Besides auxin biosynthesis proteins, many auxin transport proteins were also found to be dual localized. For instance, auxin efflux carriers PIN1-PIN4 and PIN7 are localized in the plasma membrane, whereas PIN5 and PIN8 are ER localized [35,36]. It was suggested that the different localization of PIN5 mediates auxin transport from cytosol into ER, which contributes to cellular auxin homeostasis [37].

### 3.5. Involvement of Auxin Biosynthesis Genes in Abiotic Stress

Auxin plays an important role in modulating multiple aspects of plant development growth and responses to environmental stimulants. Auxin-biosynthesis-related genes, including *TARs* and *YUCs*, were shown to be involved in shade avoidance, drought resistance, seed development as well as plant architecture regulation. In Arabidopsis, expression levels of *YUC7*, *9*, *10* and *11* genes were upregulated under dehydration condition [38]. Activation-tagged mutant *yuc7-1D* with higher total auxin levels confers drought resistance [38]. Potato and poplar plants overexpressing the Arabidopsis *YUC6* gene display auxin-overproduced phenotypes and enhanced drought tolerance [39,40]. In contrast, the *yuc1/yuc2/yuc6* triple mutant contained a lower auxin level and decreased stress tolerance [41]. In our study, overexpressed *BnaYUC6a*, both in Arabidopsis and oilseed rape, showed typical auxin overproduction alternation and conferred high drought resistance. Interestingly, we found that the *BnaYUC4a* gene is also related to drought resistance, and *BnaYUC4a-*overexpressing lines have similar phenotypes to BnaYUC6a-ox lines. All these results reveal the direct link between endogenous auxin levels and drought resistance.

### 3.6. Auxin Biosynthesis Genes Play Crucial Roles in Plant Development

We identified one candidate gene related to branch angle regulation in *B. napus* via QTL-seq previously [26]. Transgenic plants overexpressing the candidate gene *BnaYUC6a* decreased the branch angle both in Arabidopsis and oilseed rape (Figure 5). The overexpression of *BnaYU6a* also caused flowering time delays. Mutations of one *YUC* gene in peas reduced leaf vein density and caused aberrant placement of free-ending veinlets [42]. It has been shown that *AtYUC6* delayed leaf senescence by activating redox-signaling-related genes [43,44]. Therefore, *YUC6* and other auxin biosynthesis genes are involved in different aspects of the plant developmental process. 

Mutants of *TAA1* gene (*sav3/taa1*) reduce shade-induced leaf hyponasty and petiole elongation, suggesting that *TAA1* is important for shade avoidance responses [14]. The yuc1yuc4 double mutant has a similar phenotype to the sav3/taa1 mutant in shaded condition [11]. In addition, the Arabidopsis *taa1* and *tar2* double mutant was a dwarf, with reduced vasculature and sterile flowers [9]. The orthologs of *TAA1*/*TAR1*/*TAR2* genes in maize (*VANISHING TASSEL* and *ZmVT2*) and rice (*FISH BONE* and *OsFIB*) were also crucial for plant development. Unlike single mutations of *TAA1*, which cause subtle morphological phenotypes in Arabidopsis, *Zmvt2* mutants cause dramatic effects on vegetative and reproductive development [16]. The loss of function in *OsFIB* results in decreased IAA levels and leads to abnormal vascularity, small panicles, abnormal organ identity and root development defects [17]. We showed that *B. napus* plants with four copies of the *TAA1* homolog simultaneously mutated and caused dramatic development defects, such as dwarfism, very small leaves and flower buds and low seed setting. However, mutations of three copies of the *TAA1* homolog caused moderate developmental defects. Taken together, *TAR* genes are redundant in the *B. napus* genome and play a pivotal role in plant development and growth.

## 4. Materials and Methods

### 4.1. Identification and Annotation of YUC Genes in the B. napus Genome

We downloaded the HMM profile of FMO-like domains (PF00743) in the Pfam database (http://pfam.xfam.org/, accessed on 7 January 2019), which was used to search the genome database of *B. napus* (http://www.genoscope.cns.fr/brassicanapus/, accessed on 1 January 2020) using the HHMER program. All non-redundant sequences were submitted to Interpro (http://www.ebi.ac.uk/interpro, accessed on 7 January 2019) to confirm the presence of the FMO domain. Sequences without complete FMO domains were excluded. Arabidopsis *TAR* gene sequences were used as a query to perform BLASTP against the *B. napus* genome. *YUC* and *TAR* genes in rice and Arabidopsis were downloaded from the rice genome project (http://rice.plantbiology.msu.edu/, accessed on 15 June 2018) and TAIR (http://www.arabidopsis.org/, accessed on 15 June 2018) database. *YUC* gene accession numbers in the *B. napus* genome database were ascertained according to phylogenetic analysis. The nomenclature of putative *YUC* and *TAR* genes in *B. napus* was in accordance with the homologous gene IDs in Arabidopsis. The exon/intron structure of each *BnaYUC* gene was displayed in the Gene Structure Display Server program (http://gsds.cbi.pku.edu.cn/index.php, accessed on 17 June 2018) by comparing the coding sequence and genomic sequence. Multiple sequence alignment of the YUC protein sequences of *Oryza sativa*, *Arabidopsis thaliana* and *Brasscia napus* was performed using ClustalX2.0 with the default parameters [45]. Phylogenetic trees were constructed in MEGA6.0 software using the neighbor-joining (NJ) method with 1000 bootstrap replications [46].

### 4.2. Plant Materials and Different Treatments

To assess the influence of chemicals on the growth of oilseed rape seedlings, seeds were sown in 1/2 MS medium with or without Yucasin or kynurenine. The seedlings were then placed at 22 °C with a 16 h light/8 h dark cycle for five days. Hypocotyls and root lengths were then measured. To analyze gene expression patterns under different treatment, seeds were germinated and cultured on gauze with water for four days. Then, the auxin biosynthesis inhibitor (Yucasin and kynurenine), or ABA and PEG6000, was added to the water. Seedlings over ground parts or roots were harvested according to different time courses. To analyze gene expression patterns under light and dark conditions after five days, cotyledons, hypocotyls and roots were harvested and used for RNA extraction and IAA quantification. To analyze the gene expression response to different temperatures, seedlings were grown at 22 °C with a 16 h light/8 h dark cycle for four days and were then transferred to 29 °C. Seedlings over ground parts or roots were harvested according to different time courses.

### 4.3. RNA Extraction and Semi-Quantitative PCR

The total RNAs were extracted with TransZol reagent (TransGen, Beijing, China). Reverse transcription was performed according to the instruction of FastQuant RT Kit (Tiangen, Beijing, China). RT-PCR was performed as described previously using the primers listed in Table S1. The expression levels of the **actin** gene in *B. napus* were used to standardize the RNA samples for each RT-PCR. The reaction was conducted using the following program: 5 min at 95 °C, 35 cycles of 30 s at 95 °C, 40 s at 56 °C and 1 min at 72 °C.

### 4.4. IAA Quantification

Three tissue samples of leaves, stems and axillary shoots were used for IAA quantification. Samples were prepared, and mixtures of compounds were separated by HPLC (Agilent 1200) and were analyzed using a hybrid triple quadrupole/linear ion trap mass spectrometer (ABI 4000 Q-Trap, Applied Biosystems, Foster City, CA, USA). IAA was quantified according to a method described previously [41].

### 4.5. Genetic Transformation

To construct *YUC* overexpression in transgenic plants in Arabidopsis, open reading frames (ORFs) of *YUC6* (*BnaA06g37430D*) and *YUC4a* (*BnaA06g37430D*) were cloned into a PBI121S vector using the B*am*HI restriction enzyme. The constructs were introduced into *Agrobacterium tumefaciens* (GV3101). Genetic transformations were performed via the floral-dip transformation method. Transgenic plants were grown under a 16 h/8 h light–dark cycle at 23 °C in a growth chamber.

Genetic transformations in *B. napus* mediated by *A. tumefaciens* were performed as described previously [47]. Semi-winter-type oilseed rape Zhongshuang 6 was selected as the transgenic recipient. In brief, seeds were put in 75% ethanol for 1 min and were then sterilized in a 1.5% mercuric chloride solution for 15 min. After germination in darkness for 5 days, etiolated hypocotyls were cut into 5–8 mm segments and were soaked in Agrobacterium liquid media for 0.5 h. Hypocotyls were transferred to a co-culture medium for 2 days and then to a selection medium for about 3 weeks. Callus was transferred to a regeneration medium and was replicated 3–4 times. Regenerated seedlings were then moved to a root medium, and plantlets with roots were transplanted into a growth chamber. All medium components were prepared as described previously [47].

### 4.6. Expression and Purification of Recombinant Proteins in E. coli 

The coding regions of *BnaYUCs* and *BnaTARs* were amplified from Zhongshuang 6 cDNA by PCR using gene-specific primers. The *AtYUC* and *AtTAR* genes were amplified from Arabidopsis. The PCR products of these genes were cloned into the pCold-TF vector, which harbors an HSP70 (heat shock protein 70) and His-tag. The expression plasmid was transformed into *Escherichia coli* strain BL21 (DE3). Protein expression was induced at 16 °C after adding 0.5 mM of IPTG and was then purified using His-tag beads (GE Healthcare, Uppsala, Sweden).

### 4.7. Enzyme Activity Assay

The enzyme activity of YUC and TAR recombinant proteins was mostly carried out according to previous methods [11,14]. For YUC activity analysis, 20 μg of proteins was added and incubated at 30 °C for 15 min to 4 h. The reaction buffer contained 2 mM of IPA, 50 mM of potassium phosphate and 10 mM of NADPH. The IPA was added to the reaction buffer before incubation at 30 °C. The HSP70 tag was used as a control reaction instead of YUC recombinant proteins. For the TAR activity analysis, the reaction was performed at 55 °C for 10 min according to a method described previously [48]. The enzymatic activity of purified His-TAR and His-YUC was determined by ultrafast liquid chromatography/electrospray ionization/tandem mass spectrometry system, as previously described [49].

### 4.8. Subcellular Localization Analysis

Full length coding regions of *YUC* and *TAR* in *B. napus* were amplified from the cDNA of Zhongshuang 11 variety. The sequences were then fused to the coding regions of GFP, driven by a 35S promoter in a pM999 vector. RFP-HDEL was used as a marker, since it was reported to localize at the endoplasmic reticulum [50]. The 35S:GFP-YUCs or 35S:GFP-TARs were co-transformed with 35S:RFP-HDEL and were transiently expressed in Arabidopsis protoplasts by treating with polyethylene glycol [51]. After 12–16 h of transformation, the florescence signal was observed using a con-focal microscope.

### 4.9. Data Analysis

Significant differences between the control and transgenic plants were analyzed by a pair-wise *t*-test in the Excel (Microsoft) program.

## 5. Conclusions

Our results reveal that the auxin two-step biosynthesis pathway is conserved with expanded numbers of *BnaYUC* and *BnaTAR* genes in allotetraploid *B. napus*. Different homoeologs of *BnaYUC* and *BnaTAR* may be divergent according to sequence and expression variations. The modulated expression of auxin biosynthesis genes can improve abiotic stress resistance and decrease branch angles. Auxin biosynthesis genes in *B. napus* play a vital role in plant developmental processes and can be utilized to improve abiotic stress resistance and plant architecture.

## Data Availability

This study did not report any data.

## Supplementary Materials

The following supporting information can be downloaded at: https://www.mdpi.com/article/10.3390/ijms232415600/s1.
