# Supplementary material for: Auxin Biosynthesis Genes in Allotetraploid Oilseed Rape Are Essential for Plant Development and Response to Drought Stress
Source: Int J Mol Sci. 2022 Dec 9;23(24):15600. doi: 10.3390/ijms232415600 (PMC9778849; doi:10.3390/ijms232415600)
Supplement: Supplementary file 1 [file ijms-23-15600-s001.zip › Figures.pptx]

## Slide 1
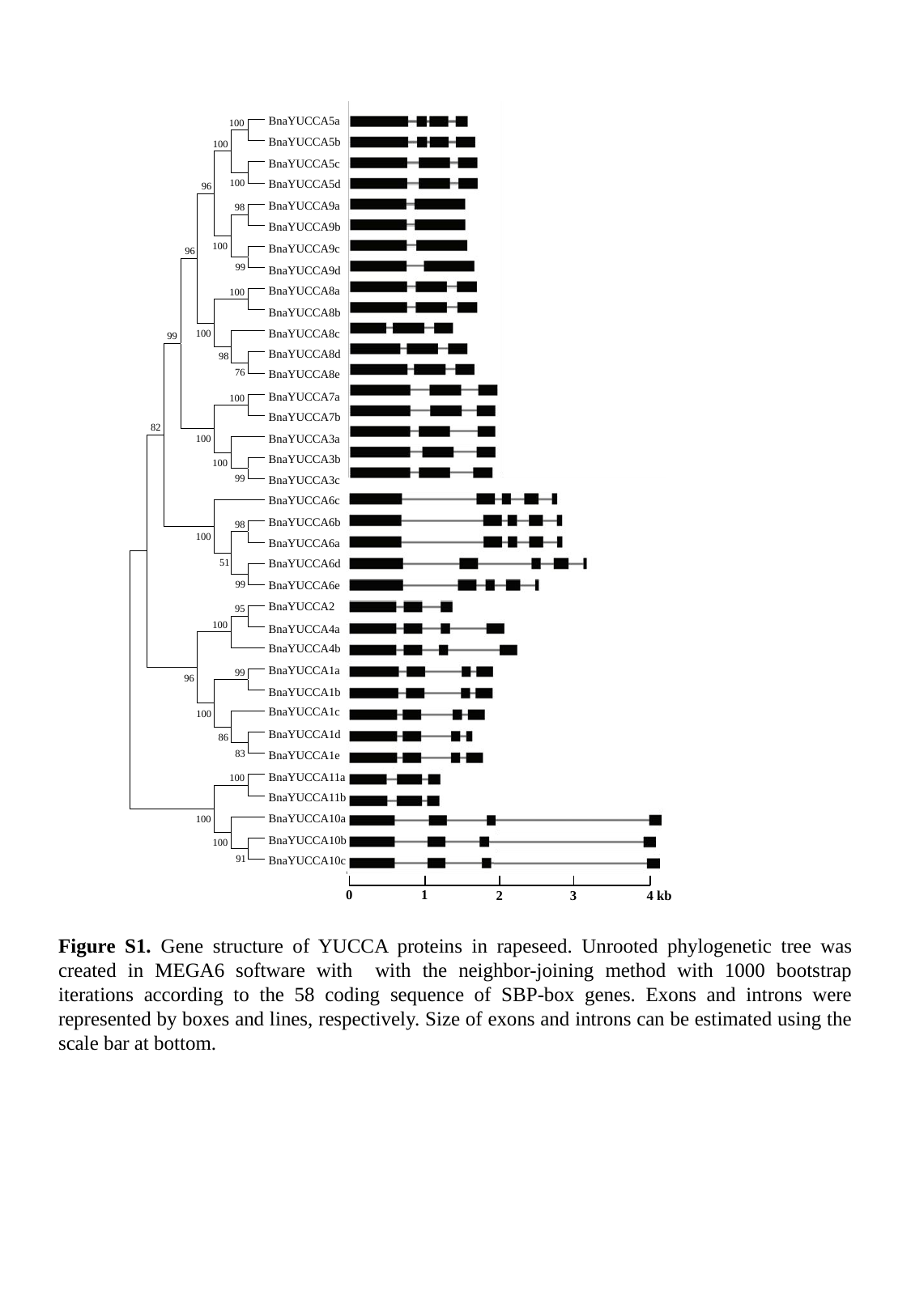

BnaYUCCA5a
 BnaYUCCA5b
 BnaYUCCA5c
 BnaYUCCA5d
 BnaYUCCA9a
 BnaYUCCA9b
 BnaYUCCA9c
 BnaYUCCA9d
 BnaYUCCA8a
 BnaYUCCA8b
 BnaYUCCA8c
 BnaYUCCA8d
 BnaYUCCA8e
 BnaYUCCA7a
 BnaYUCCA7b
 BnaYUCCA3a
 BnaYUCCA3b
 BnaYUCCA3c
 BnaYUCCA6c
 BnaYUCCA6b
 BnaYUCCA6a
 BnaYUCCA6d
 BnaYUCCA6e
 BnaYUCCA2
 BnaYUCCA4a
 BnaYUCCA4b
 BnaYUCCA1a
 BnaYUCCA1b
 BnaYUCCA1c
 BnaYUCCA1d
 BnaYUCCA1e
 BnaYUCCA11a
 BnaYUCCA11b
 BnaYUCCA10a
 BnaYUCCA10b
 BnaYUCCA10c
100
100
100
96
98
100
96
99
100
100
99
98
76
100
82
100
100
99
98
100
51
99
95
100
99
96
100
86
83
100
100
100
91
0
1
2
3
4 kb
Figure S1. Gene structure of YUCCA proteins in rapeseed. Unrooted phylogenetic tree was created in MEGA6 software with with the neighbor-joining method with 1000 bootstrap iterations according to the 58 coding sequence of SBP-box genes. Exons and introns were represented by boxes and lines, respectively. Size of exons and introns can be estimated using the scale bar at bottom.

## Slide 2
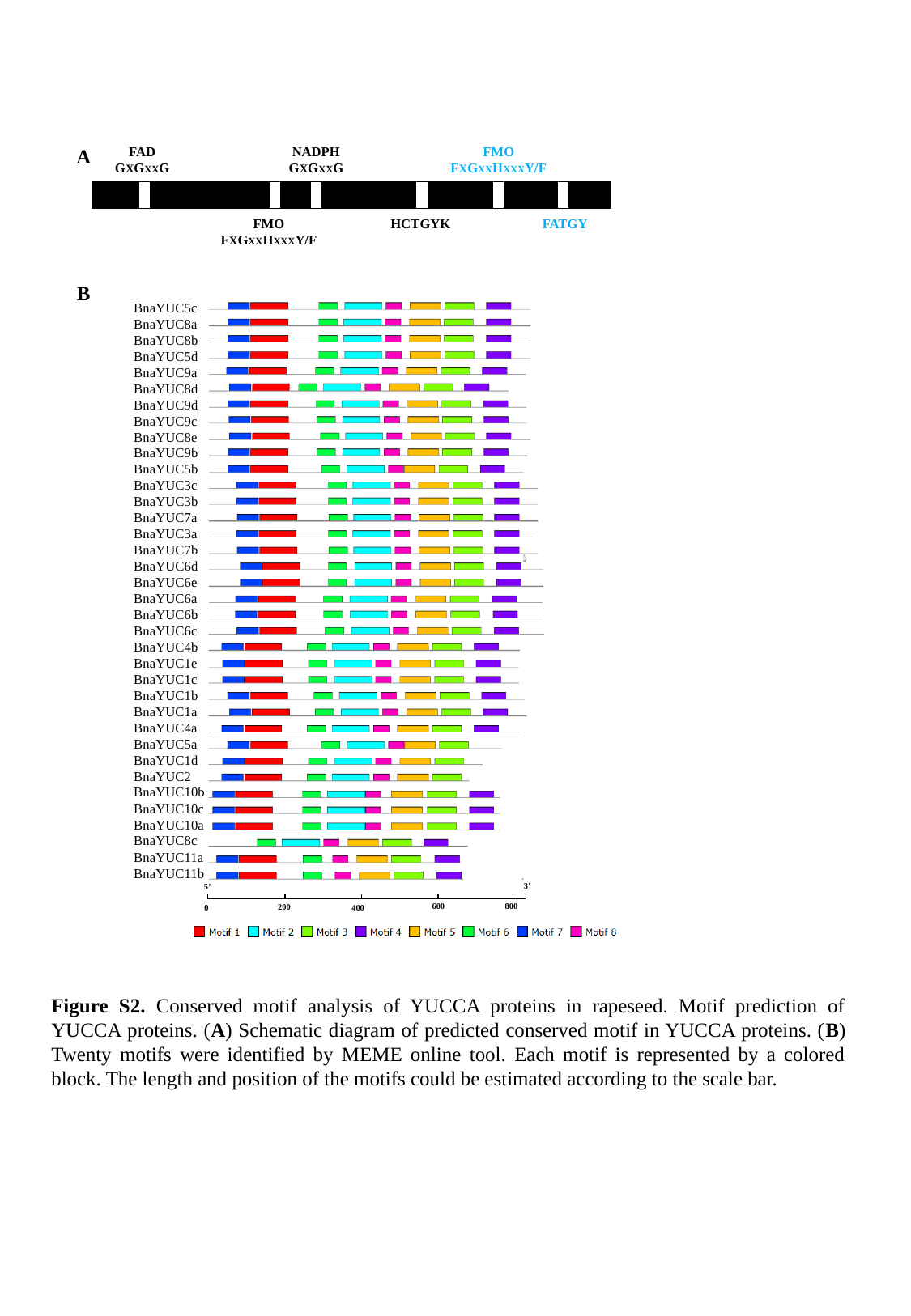

A
FAD
GXGXXG
NADPH
GXGXXG
FMO
FXGXXHXXXY/F
FMO
FXGXXHXXXY/F
HCTGYK
FATGY
B
BnaYUC5c
BnaYUC8a
BnaYUC8b
BnaYUC5d
BnaYUC9a
BnaYUC8d
BnaYUC9d
BnaYUC9c
BnaYUC8e
BnaYUC9b
BnaYUC5b
BnaYUC3c
BnaYUC3b
BnaYUC7a
BnaYUC3a
BnaYUC7b
BnaYUC6d
BnaYUC6e
BnaYUC6a
BnaYUC6b
BnaYUC6c
BnaYUC4b
BnaYUC1e
BnaYUC1c
BnaYUC1b
BnaYUC1a
BnaYUC4a
BnaYUC5a
BnaYUC1d
BnaYUC2
BnaYUC10b
BnaYUC10c
BnaYUC10a
BnaYUC8c
BnaYUC11a
BnaYUC11b
3’
5’
800
600
200
400
0
Figure S2. Conserved motif analysis of YUCCA proteins in rapeseed. Motif prediction of YUCCA proteins. (A) Schematic diagram of predicted conserved motif in YUCCA proteins. (B) Twenty motifs were identified by MEME online tool. Each motif is represented by a colored block. The length and position of the motifs could be estimated according to the scale bar.

## Slide 3
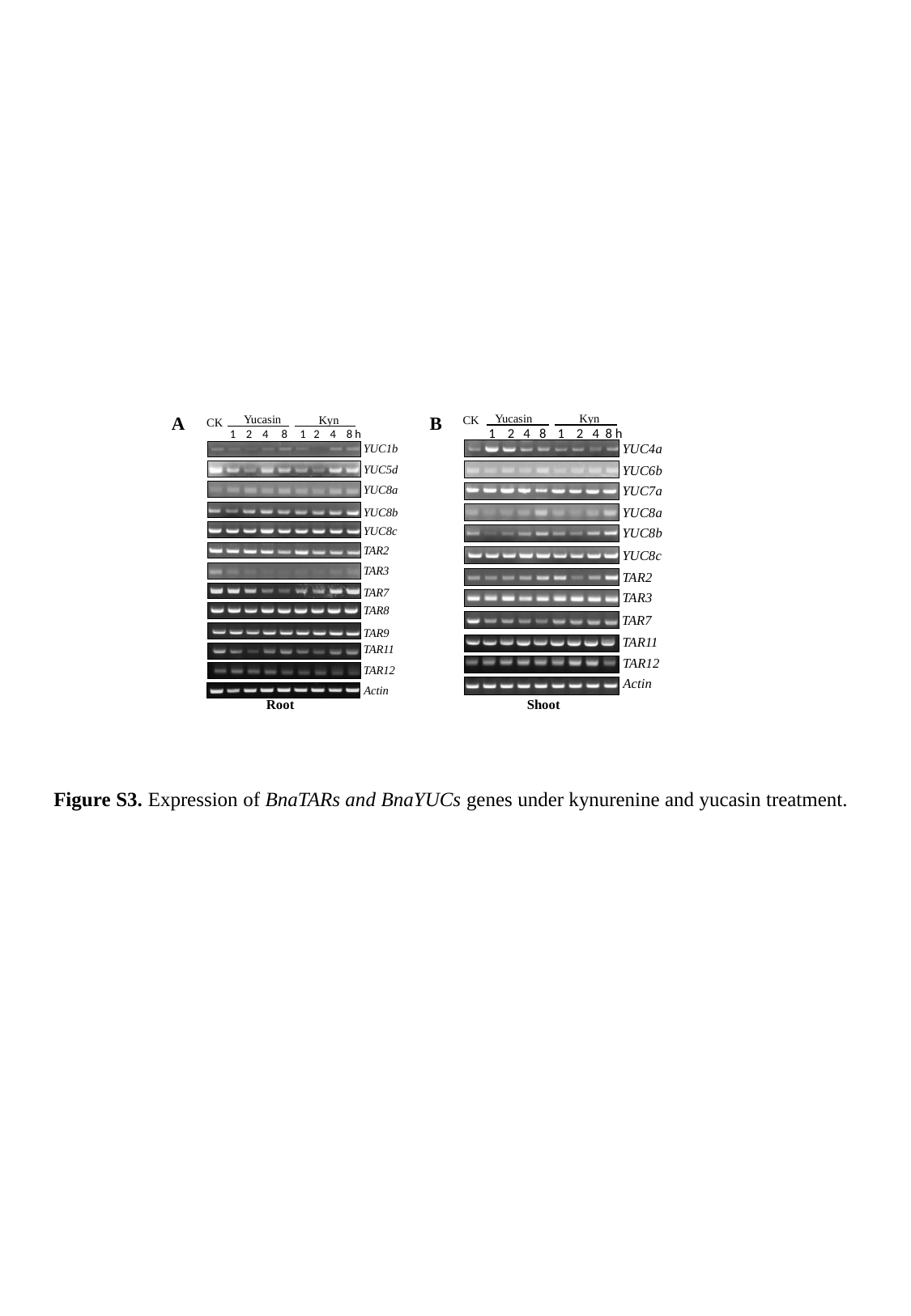

Kyn
Yucasin
CK
1 2 4 8 h
1 2 4 8
YUC4a
YUC6b
YUC7a
YUC8a
YUC8b
YUC8c
TAR2
TAR3
TAR7
TAR11
TAR12
Actin
Shoot
A
B
Yucasin
Kyn
CK
1 2 4 8 h
1 2 4 8
YUC1b
YUC5d
YUC8a
YUC8b
YUC8c
TAR2
TAR3
TAR7
TAR8
TAR9
TAR11
TAR12
Actin
Root
Figure S3. Expression of BnaTARs and BnaYUCs genes under kynurenine and yucasin treatment.

## Slide 4
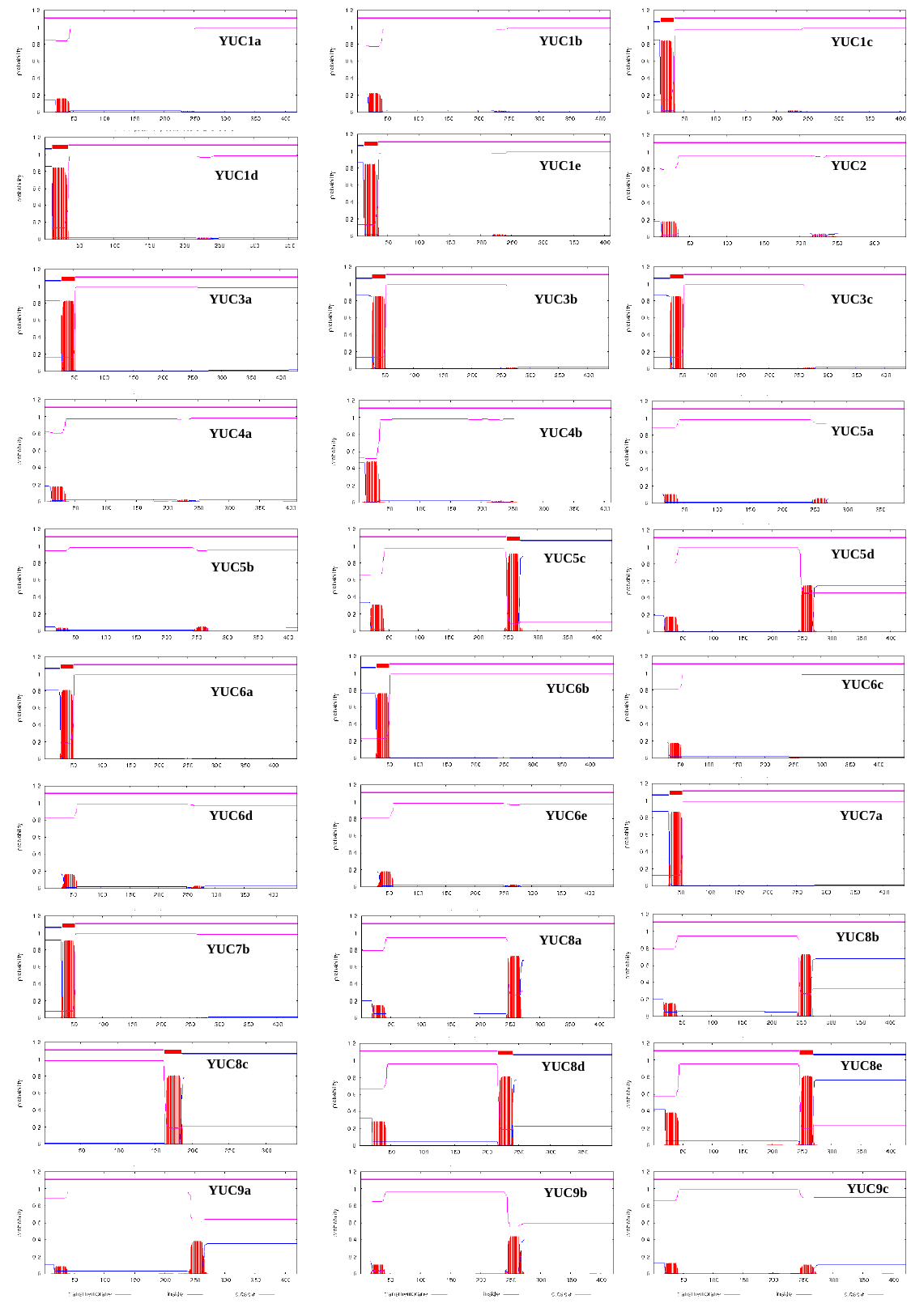

YUC1b
YUC1a
YUC1c
YUC1e
YUC2
YUC1d
YUC3a
YUC3b
YUC3c
YUC5a
YUC4b
YUC4a
YUC5d
YUC5c
YUC5b
YUC6c
YUC6b
YUC6a
YUC6d
YUC6e
YUC7a
YUC8b
YUC8a
YUC7b
YUC8c
YUC8e
YUC8d
YUC9c
YUC9a
YUC9b

## Slide 5
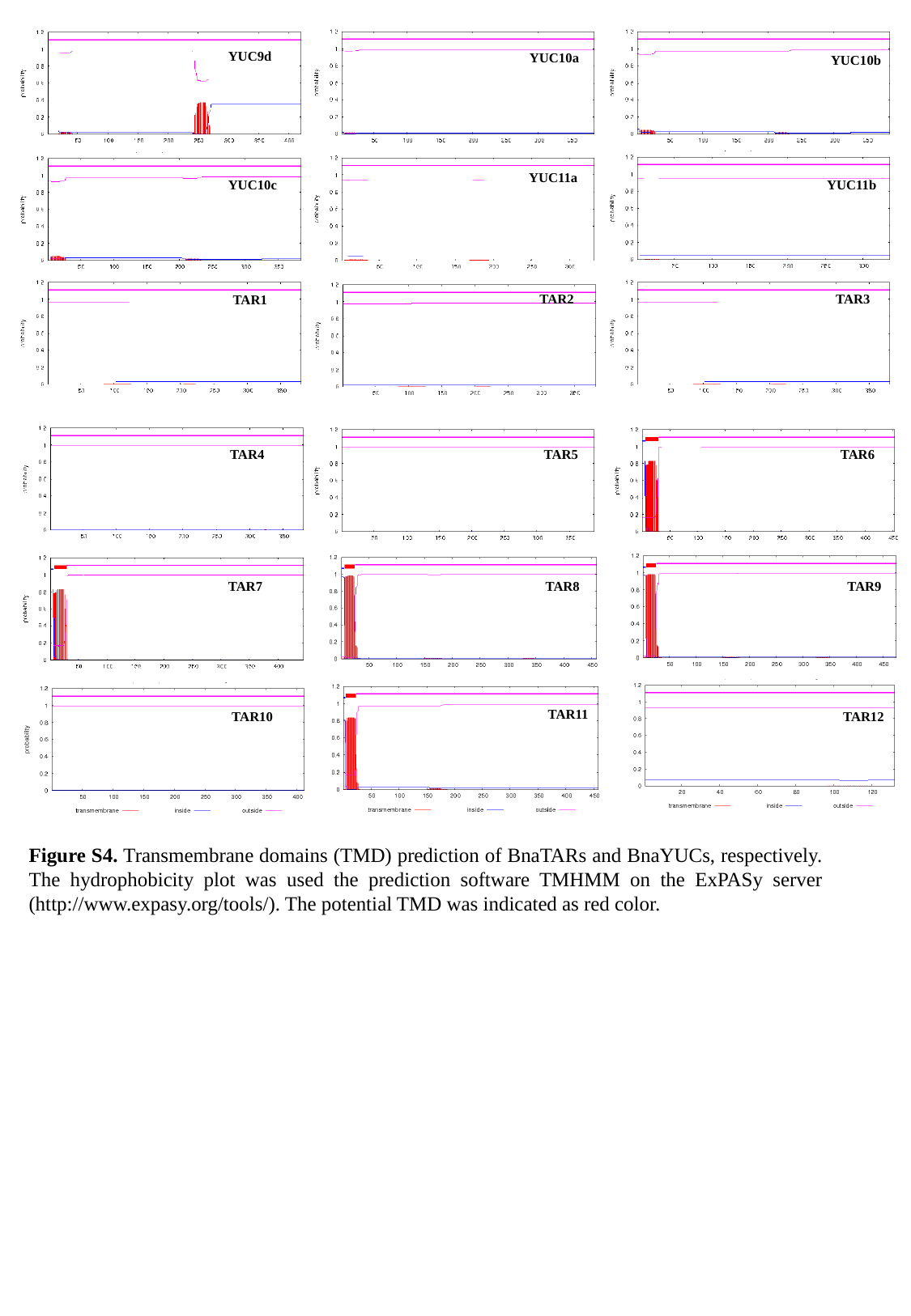

YUC9d
YUC10a
YUC10b
YUC11a
YUC10c
YUC11b
TAR2
TAR3
TAR1
TAR5
TAR6
TAR4
TAR7
TAR8
TAR9
TAR11
TAR10
TAR12
Figure S4. Transmembrane domains (TMD) prediction of BnaTARs and BnaYUCs, respectively. The hydrophobicity plot was used the prediction software TMHMM on the ExPASy server (http://www.expasy.org/tools/). The potential TMD was indicated as red color.

## Slide 6
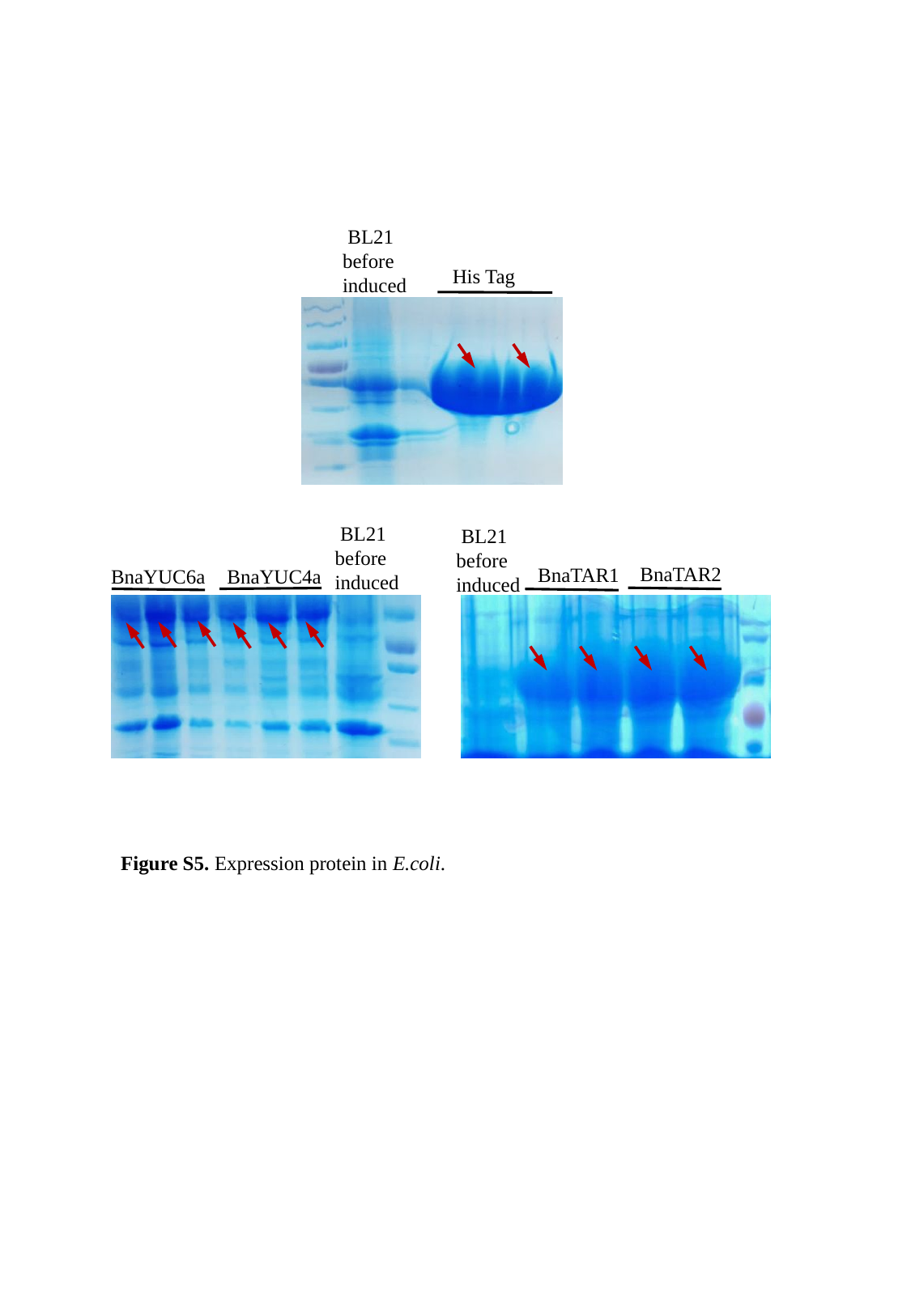

BL21
before
induced
His Tag
 BL21
before
induced
BnaYUC6a
BnaYUC4a
 BL21
before
induced
BnaTAR2
BnaTAR1
Figure S5. Expression protein in E.coli.

## Slide 7
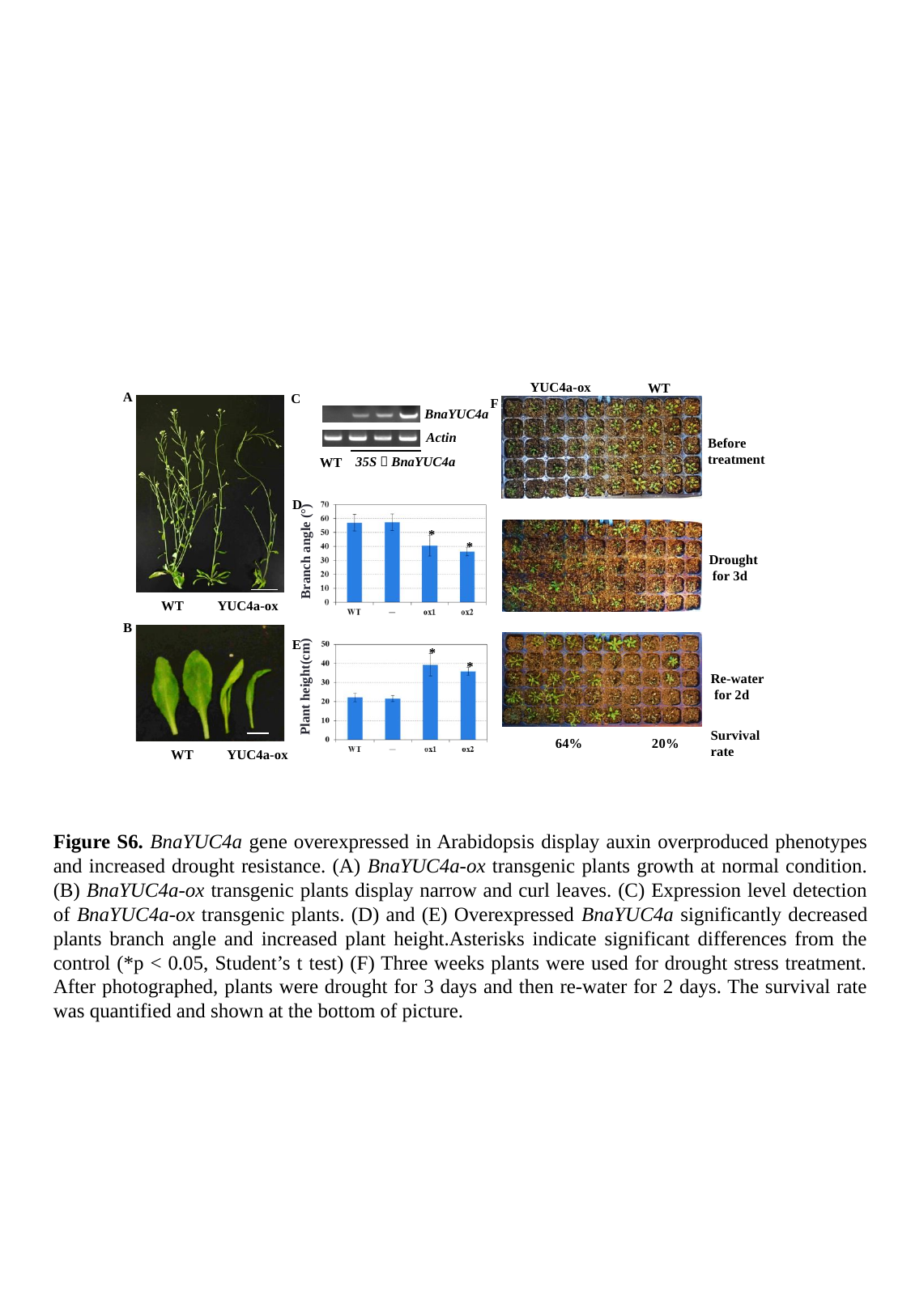

YUC4a-ox
WT
A
C
F
BnaYUC4a
Actin
Before treatment
35S：BnaYUC4a
WT
Branch angle (°)
D
*
*
Drought
 for 3d
WT
YUC4a-ox
B
Plant height(cm)
E
*
*
Re-water
 for 2d
Survival rate
64%
20%
WT
YUC4a-ox
Figure S6. BnaYUC4a gene overexpressed in Arabidopsis display auxin overproduced phenotypes and increased drought resistance. (A) BnaYUC4a-ox transgenic plants growth at normal condition. (B) BnaYUC4a-ox transgenic plants display narrow and curl leaves. (C) Expression level detection of BnaYUC4a-ox transgenic plants. (D) and (E) Overexpressed BnaYUC4a significantly decreased plants branch angle and increased plant height.Asterisks indicate significant differences from the control (*p < 0.05, Student’s t test) (F) Three weeks plants were used for drought stress treatment. After photographed, plants were drought for 3 days and then re-water for 2 days. The survival rate was quantified and shown at the bottom of picture.

## Slide 8
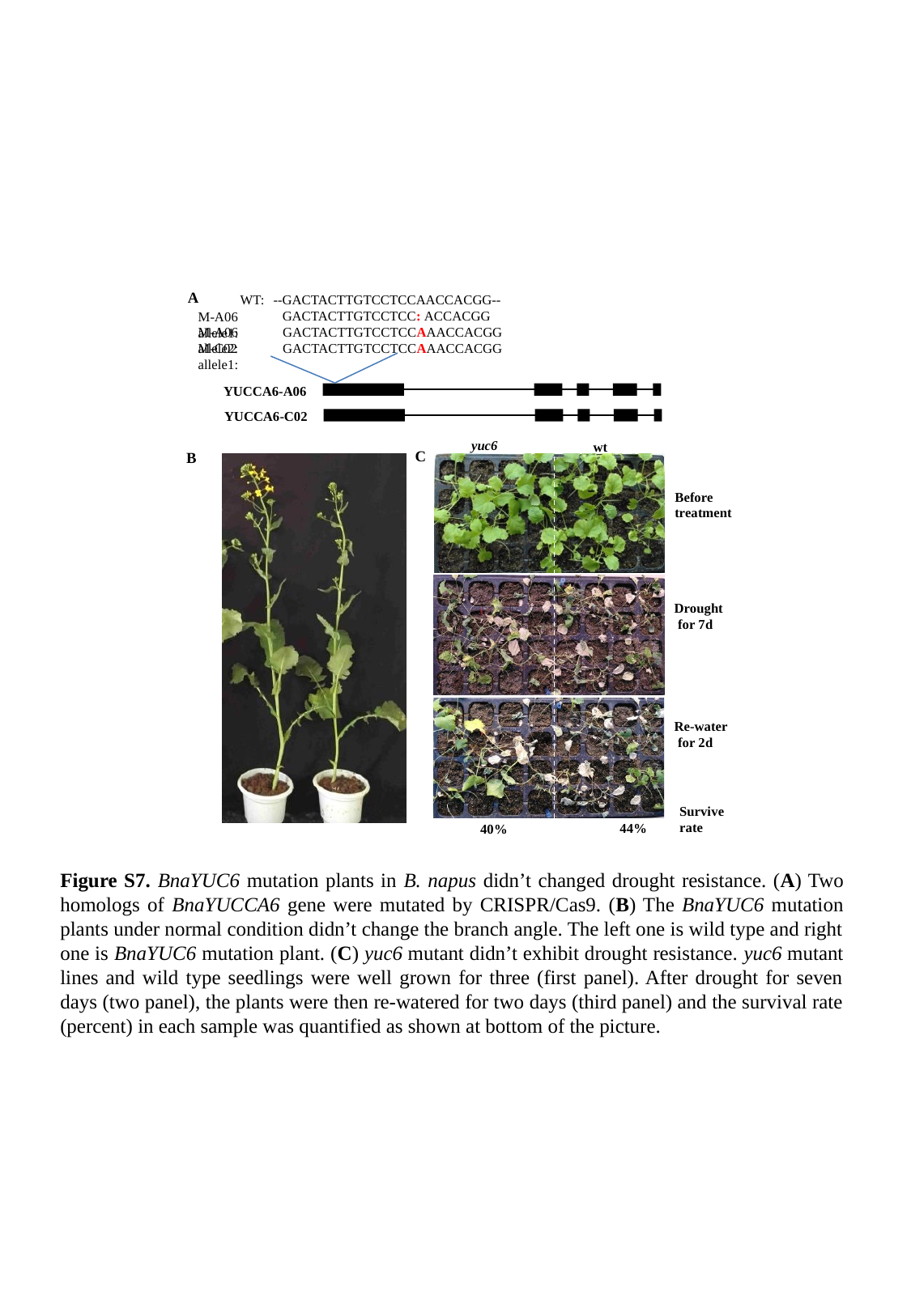

A
--GACTACTTGTCCTCCAACCACGG--
WT:
GACTACTTGTCCTCC: ACCACGG
M-A06 allele1:
GACTACTTGTCCTCCAAACCACGG
M-A06 allele2:
M-C02 allele1:
GACTACTTGTCCTCCAAACCACGG
YUCCA6-A06
YUCCA6-C02
yuc6
wt
C
B
Before treatment
Drought
 for 7d
Re-water
 for 2d
Survive rate
44%
40%
Figure S7. BnaYUC6 mutation plants in B. napus didn’t changed drought resistance. (A) Two homologs of BnaYUCCA6 gene were mutated by CRISPR/Cas9. (B) The BnaYUC6 mutation plants under normal condition didn’t change the branch angle. The left one is wild type and right one is BnaYUC6 mutation plant. (C) yuc6 mutant didn’t exhibit drought resistance. yuc6 mutant lines and wild type seedlings were well grown for three (first panel). After drought for seven days (two panel), the plants were then re-watered for two days (third panel) and the survival rate (percent) in each sample was quantified as shown at bottom of the picture.

## Slide 9
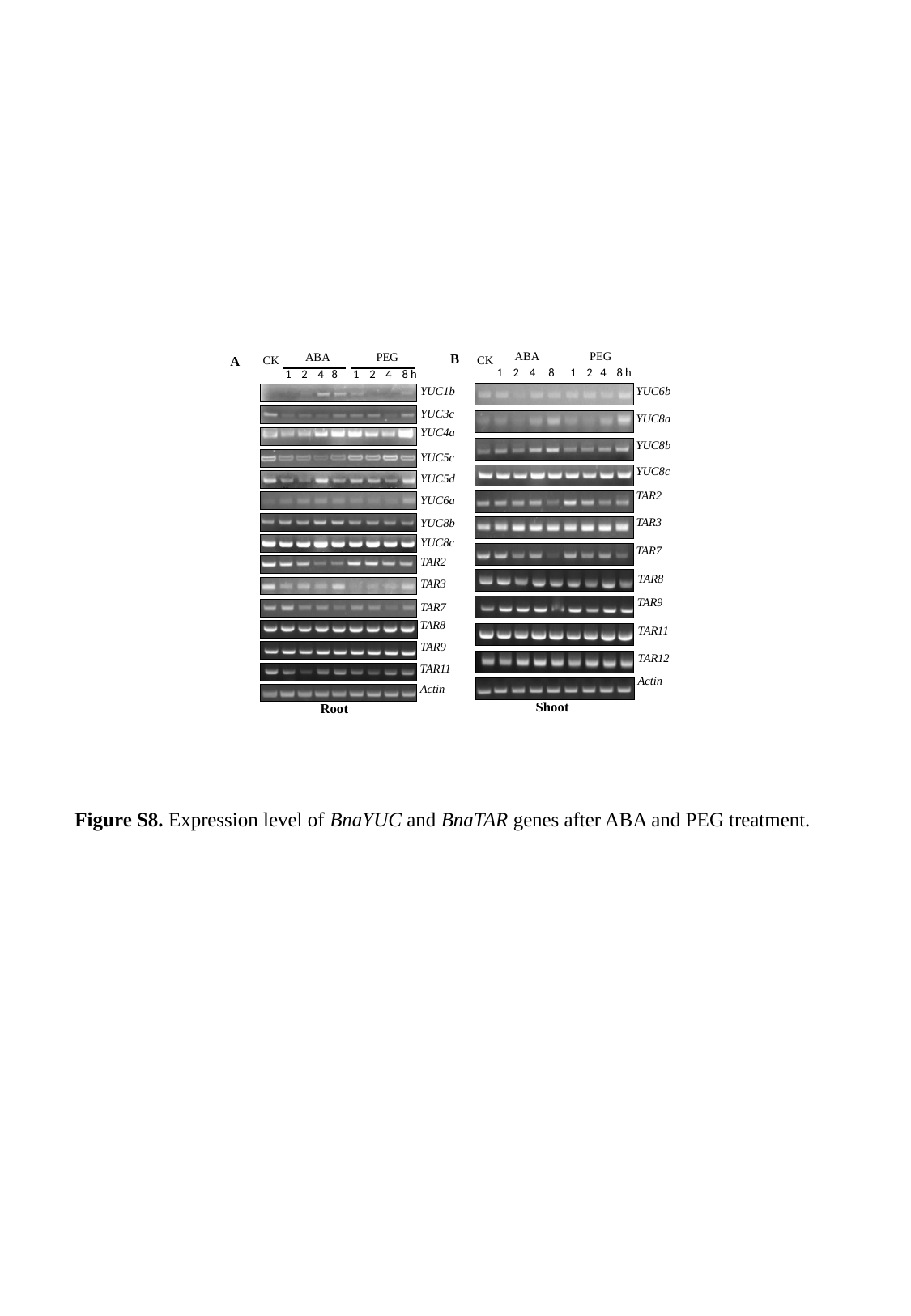

PEG
ABA
CK
1 2 4 8 h
1 2 4 8
YUC6b
YUC8a
YUC8b
YUC8c
TAR2
TAR3
TAR7
TAR8
TAR9
TAR11
TAR12
Actin
PEG
ABA
B
A
CK
1 2 4 8 h
1 2 4 8
YUC1b
YUC3c
YUC4a
YUC5c
YUC5d
YUC6a
YUC8b
YUC8c
TAR2
TAR3
TAR7
TAR8
TAR9
TAR11
Actin
Shoot
Root
Figure S8. Expression level of BnaYUC and BnaTAR genes after ABA and PEG treatment.

## Slide 10
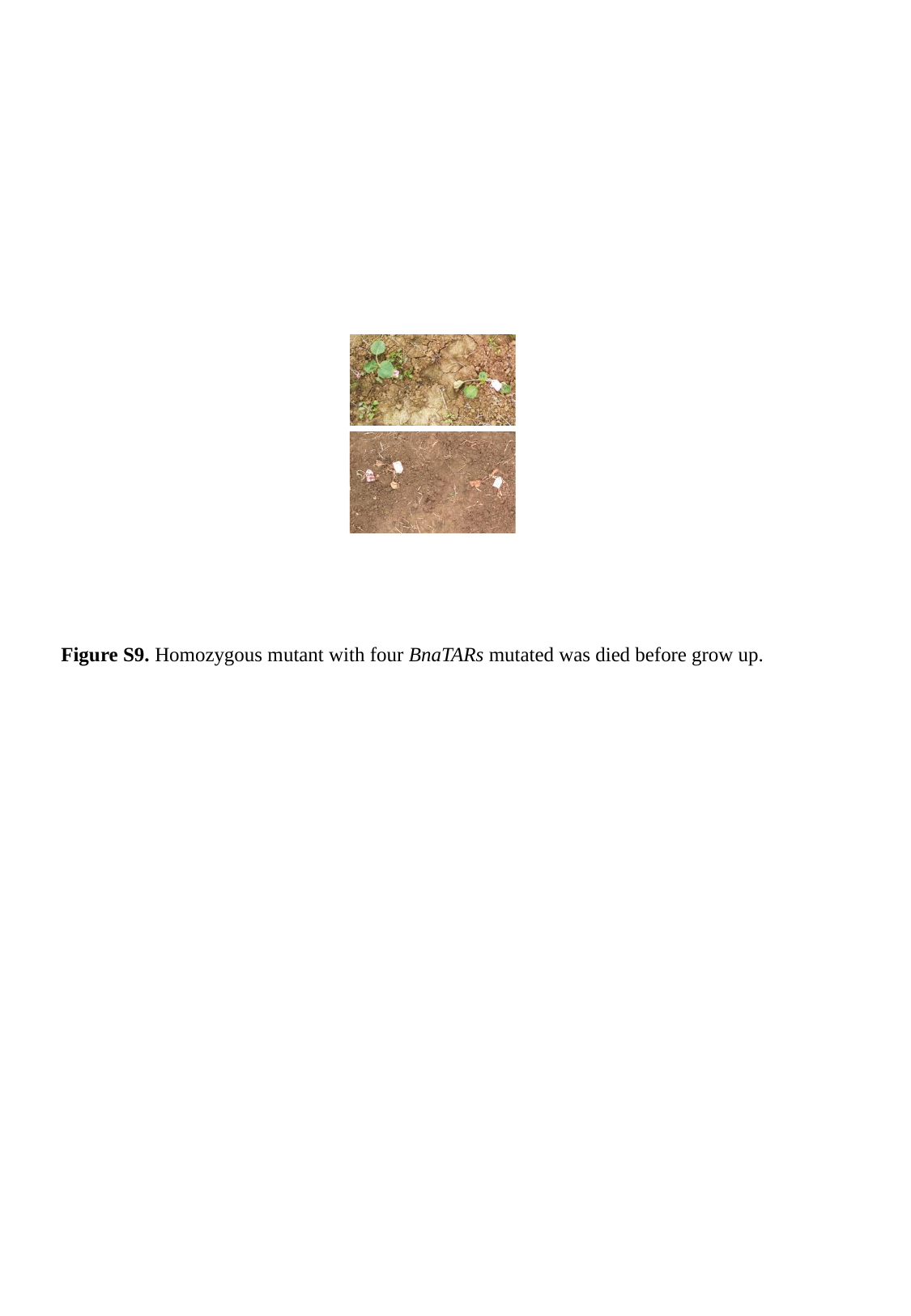

Figure S9. Homozygous mutant with four BnaTARs mutated was died before grow up.

## Slide 11
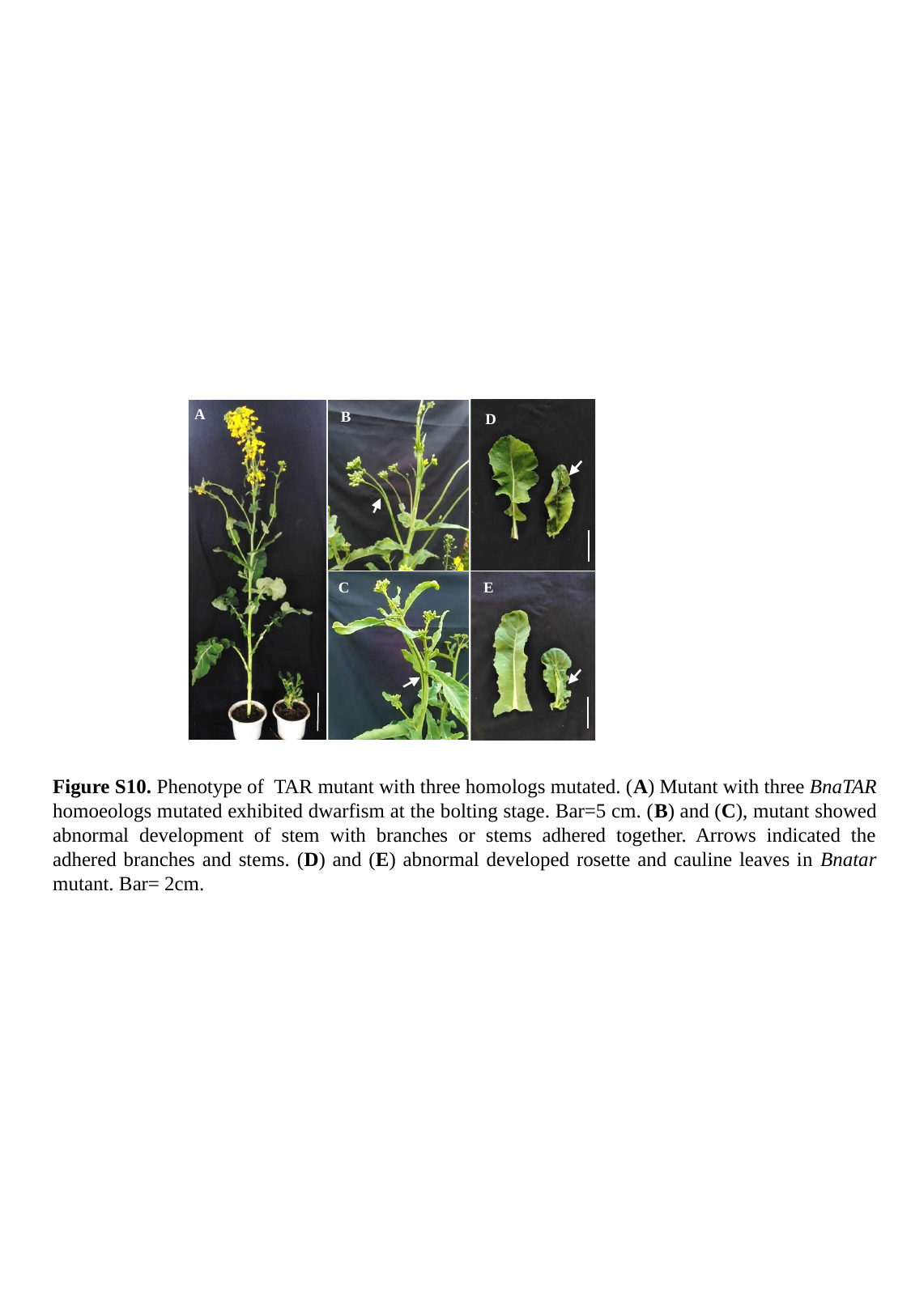

A
B
D
G
H
C
E
Figure S10. Phenotype of TAR mutant with three homologs mutated. (A) Mutant with three BnaTAR homoeologs mutated exhibited dwarfism at the bolting stage. Bar=5 cm. (B) and (C), mutant showed abnormal development of stem with branches or stems adhered together. Arrows indicated the adhered branches and stems. (D) and (E) abnormal developed rosette and cauline leaves in Bnatar mutant. Bar= 2cm.
